# Discovery and characterization of an FAD-dependent glucose 6-dehydrogenase

**DOI:** 10.1016/j.jbc.2025.108189

**Published:** 2025-01-13

**Authors:** Takahiro Fujii, Michinari Honda, Wataru Fujii, Yoshimi Shimada, Michiki Takeuchi, Jun Ogawa

**Affiliations:** 1 Ikeda Food Research Co., Ltd, Fukuyama Hiroshima, Japan; 2 Division of Applied Life Sciences, Graduate School of Agriculture, Kyoto University, Kyoto, Japan; 3 Industrial Microbiology, Graduate School of Agriculture, Kyoto University, Kyoto, Japan; 4 Faculty of Molecular Chemistry and Engineering, Kyoto Institute of Technology, Kyoto, Japan

**Keywords:** glucose 6-dehydrogenase, glucuronic acid, glucose, substrate specificity, flavoprotein, fungi, diabetes diagnosis, diabetes

## Abstract

Many patients with diabetes use self-measurement devices for blood glucose to understand their blood glucose levels. Most of these devices use FAD-dependent glucose dehydrogenase (FAD-GDH) to determine blood glucose levels. For this purpose, FAD-GDHs specifically oxidizing glucose among the sugars present in blood are required. Many FAD-GDHs with high substrate specificity have been reported previously; however, their substrate specificity is insufficient as they also react with xylose. Therefore, we aimed to identify FAD-GDHs without xylose reactivity. We screened and obtained a new enzyme from *Colletotrichum plurivorum* (*Cp*GDH). *Cp*GDH showed high activity to glucose in the presence of electron mediators but low activity to xylose. We prepared the glucose oxidation products using *Cp*GDH and subjected to TLC, HPLC, mass spectrometry, and NMR analyses. The results demonstrated that *Cp*GDH is a previously unknown FAD-dependent glucose 6-dehydrogenase (FAD-G6DH) that oxidizes glucose to glucuronic acid. The stoichiometric ratio of the substrate and electron mediator was 1:2, suggesting that *Cp*GDH catalyzes two-step oxidation reactions, including oxidation of primary alcohols to aldehydes and of aldehydes to carboxylic acids. We concluded that *Cp*GDH has the unique substrate-binding manner based on the result of docking simulation of *Cp*GDH with a substrate glucose. We then constructed a phylogenetic tree of carbohydrate-related flavoproteins including FAD-G6DHs, indicating that FAD-G6DHs are different from the known FAD-dependent oxidoreductases. Overall, this study is the first to report FAD-G6DHs. These results will likely contribute to the development of more accurate blood glucose sensors and further research on the metabolisms of glucosides and their metabolites.

Several saccharide-related enzymes have been identified and used to produce profitable materials (1, 2, 3) and measure the concentrations of specific molecules as biomarkers (4, 5, 6). Among these, FAD-dependent glucose dehydrogenases (FAD-GDHs) are important for human health because some self-measuring blood glucose (SMBG) devices use FAD-GDH.

Diabetes, a chronic disease characterized by hyperglycemia, can cause eye damage (7, 8) and kidney disease (9, 10) and decrease the quality of life. Many patients with diabetes need to inject insulin and improve their eating habits to decrease and control their blood glucose levels. Further, blood glucose levels must be monitored using SMBG devices to avoid the risk of hypoglycemia upon insulin injection. Hypoglycemia can cause serious brain damage and be life-threatening; therefore, accurate blood glucose sensors are essential.

SMBG devices equipped with FAD-GDH can accurately measure blood glucose levels and are used worldwide. The accuracy of SMBG devices equipped with FAD-GDH is determined based on the substrate specificity of FAD-GDH. Efficient SMBG devices require FAD-GDHs with high substrate specificity for glucose but no reactivity to sugars, such as maltose and galactose (11, 12), which are contained in blood as well as in infusion solutions. The most common FAD-GDHs are glucose 1-dehydrogenases (G1DHs), which catalyze hydroxy group oxidation at the C1-position of glucose, as shown in Fig. 1*A* (13, 14). G1DHs oxidize glucose to gluconolactone, which is hydrolyzed to gluconic acid by water. FAD-GDHs derived from *Aspergillus terreus* (15) and *Aspergillus oryzae* (16) are G1DHs, which have high specific activity and can be efficiently produced, and then are compatible for use in SMBG devices. However, the existing SMBG devices equipped with FAD-GDH may show incorrect blood glucose levels owing to the reactivity of FAD-GDHs for xylose (17). Xylose has a structure similar to that of glucose and a lower molecular weight; therefore, xylose—instead of glucose—binds to FAD-GDHs. Only a few FAD-GDHs show low reactivity to xylose. Thus, several studies have focused on improving substrate specificity using site-specific mutagenesis and have created mutated FAD-GDHs with lower reactivity for xylose; however, these mutated FAD-GDHs have low specific activity (18). Reducing the reactivity of molecules smaller than the main substrate molecule is very difficult.

**Figure 1:**
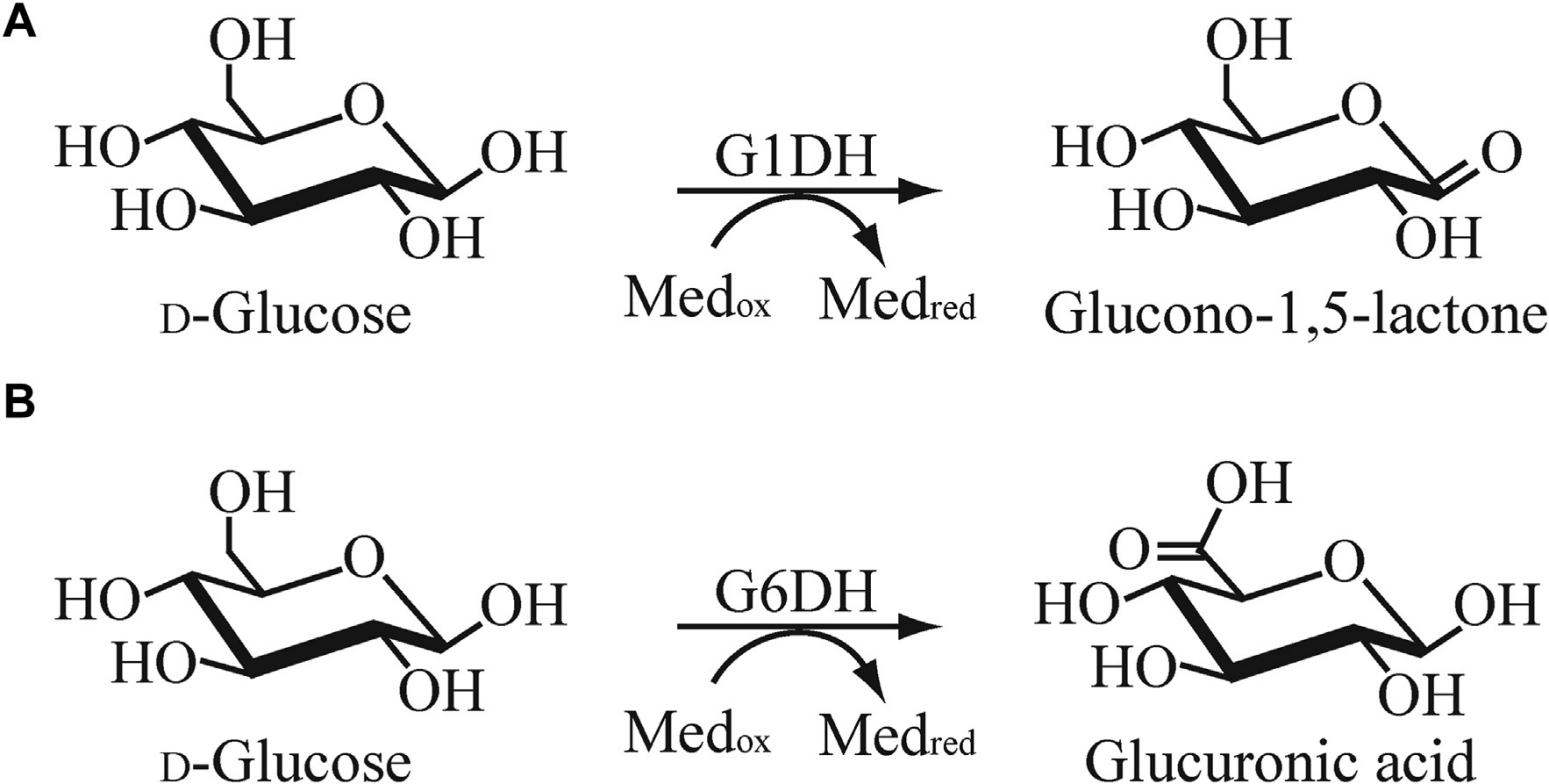
**Oxidation process of glucose 1-dehydrogenase (G1DH) and glucose 6-dehydrogenase (G6DH)**. *A*, illustration of the established C1 oxidation process mediated by G1DH and (*B*) proposed C6 oxidation pathway involving G6DH.

Therefore, in this study, we aimed to screen enzymes to obtain novel GDHs with low reactivity toward xylose, maltose, and galactose, and resulted in discovering *Cp*GDH, a novel FAD-GDH from a plant pathogenic fungus *Colletotrichum plurivorum*. In this article, we characterized *Cp*GDH and also demonstrated that *Cp*GDH is a novel naturally occurring FAD-dependent glucose 6-dehydrogenase (G6DH) catalyzing the glucose oxidation to glucuronic acid as described in Fig. 1*B via* the corresponding aldehyde intermediate.

## Results

### Discovering *Cp*GDH

**Figure 2:**
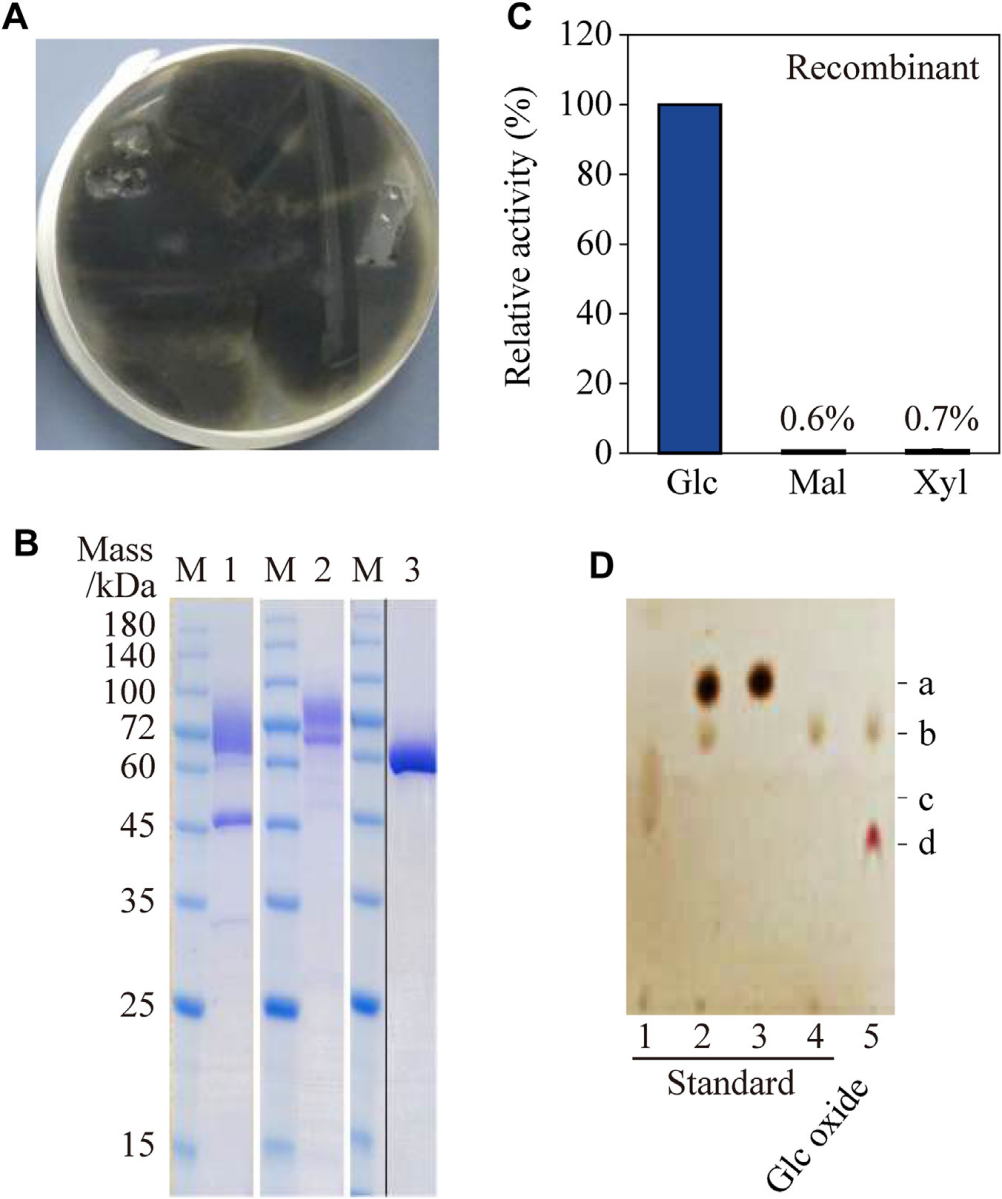
**Characterization of the glucose dehydrogenase produced by plant pathogenic fungus *Colletotrichum plurivorum* (*Cp*GDH) and the substrate specificity of the recombinant *Cp*GDH**. *A*, photograph of WT *C*. *plurivorum* cultured on an agar plate. *B*, SDS-PAGE analysis of the recombinant *Cp*GDH. LaneM, standard molecular weight marker proteins; lane1, crude recombinant *Cp*GDH; lane2, purified recombinant *Cp*GDH; lane3, deglycosylated and purified recombinant *Cp*GDH. *C*, substrate specificity of the purified recombinant *Cp*GDH. The values represent the mean ± S.D. *D*, TLC results of the glucose oxidation product produced with *Cp*GDH. Lanes 1 to 4 include the standards, whereas lane 5 represents the product of glucose oxidation obtained with *Cp*GDH. lane 1, 320 mM gluconic acid; lane 2, a mixture of 50 mM glucose and 50 mM glucuronic acid; lane 3, 50 mM glucose; and lane 4, 50 mM glucuronic acid. In the TLC plate, the spots at a, b, c, and d correspond to glucose, glucuronic acid, gluconic acid, and oxidized 1-m-PMS, respectively. 1-m-PMS, 1-methoxy-5-methylphenazinium methylsulfate; Glc, d-glucose; Mal, maltose; Xyl, d-xylose.

We did enzyme screening to obtain new GDHs that showed ignorable reactivity toward xylose, maltose, and galactose, and found candidate activity in the culture supernatant of a plant pathogenic fungus *C*. *plurivorum*. Fig. 2*A* depicts a photograph of *C*. *plurivorum* grown on a nutrient plate. We cultured the strain in a nutrient-liquid medium and collected the culture supernatant to investigate the activity and substrate specificity of the candidate enzyme, *Cp*GDH. The enzyme activity was detected toward glucose; however, almost no activity was detected for xylose and maltose (data not shown).

### Recombinant expression of *Cp*GDH and its substrate specificity

We amplified the candidate gene of *Cp*GDH from the *C*. *plurivorum* genome using primers designed based on the *gdh* sequences and expressed it in *A*. *oryzae*. The recombinant *Cp*GDH released into the culture medium was purified using hydrophobic- and anion-exchange chromatography (Table 1). Finally, the specific activity of *Cp*GDH was improved about 1.9-fold by enzyme purification. SDS-PAGE revealed two purified recombinant *Cp*GDH fragments of 60 kDa and 72 kDa (Fig. 2*B*). We also prepared recombinant *Cp*GDH, which was deglycosylated using endoglycosidase, purified, and subjected to SDS-PAGE analysis. As deglycosylated recombinant *Cp*GDH yielded a single band of 60 kDa, both the 60 kDa and 72 kDa forms of *Cp*GDH detected in the culture supernatant of the transformant were suggested to be identical with different types of glycosylation. Molecular mass of *Cp*GDH was calculated as 60 kDa with gel filtration analysis. These results indicated that recombinant *Cp*GDH existed as monomeric enzyme. To demonstrate that the recombinant *Cp*GDH does not exhibit activity against xylose, we evaluated the substrate specificity of purified recombinant *Cp*GDH without deglycosylation. Recombinant *Cp*GDH showed dehydrogenase activity toward glucose but almost no activity toward xylose and maltose (Fig. 2*C*). The WT enzyme in the culture supernatant of *C*. *plurivorum* showed dehydrogenase activity similar to that of the recombinant enzyme.

**Table 1:** Purification of *Colletotrichum plurivorum* GDH (*Cp*GDH)

Purification step	Total activity (U, × 10^5^)	Total protein (mg, × 10^3^)	Specific activity (U/mg)	Yield (%)	Purification (Fold)
Culture supernatant	3.13	4.98	62.9	-	-
Buffer exchange (desalting)	1.52	2.59	58.7	100	1.00
(NH_4_)_2_SO_4_ precipitation	1.39	2.18	63.8	91.4	1.09
Butyl chromatography	1.61	1.98	81.3	106	1.39
DEAE chromatography	1.17	1.06	110	77.0	1.87

One unit is defined as the amount of enzyme that converts 1 μmole of glucose to gluconic acid or glucuronic acid per minute. Specific activities of dehydrogenase toward glucose were determined using a 2,6-dichloroindophenol assay. After the culture supernatant was desalted and concentrated, a part of it was used as a specimen for *Cp*GDH purification.

### TLC analysis of the glucose oxidation product

As *Cp*GDH oxidized glucose but not xylose, we considered that it oxidized the hydroxy group at the C6 position and carried out a TLC analysis of the glucose oxidation product prepared using *Cp*GDH. As shown in Fig. 2*D*, glucuronic acid was only detected in the lane corresponding to the oxidation product of *Cp*GDH. Substrate glucose, gluconic acid, and other sugar oxidation products were not detected, suggesting that *Cp*GDH is a novel FAD-GDH that specifically catalyzes hydroxy group oxidation at the C6-position of glucose. Furthermore, we conducted Fehling’s test of the sugar-oxidized products obtained using *Cp*GDH to confirm that the reducing end remained intact. All of the products yielded a red precipitate (Table S1), which indicated that *Cp*GDH did not oxidize the hydroxy group at the C1-position of the sugars.

### Analysis of glucuronic acid and gluconic acid

To further determine whether the glucose oxidation product was glucuronic acid, the enzyme reaction mixture was analyzed using assay kits for d-hexuronic acid and gluconic acid, respectively; this was done after removing the electron acceptor 1-methoxy-5-methylphenazinium methylsulfate (1-m-PMS) from the reaction mixture. Neither kit responded to glucose; however, they did respond to either glucuronic acid or gluconic acid, respectively. Each kit could evaluate the concentration of glucuronic acid or gluconic acid without cross-reactivity. Glucuronic acid was detected in the reaction mixture with *Cp*GDH using a hexuronic acid assay kit, whereas gluconic acid was not detected using this kit (Table 2). In contrast, gluconic acid was detected in the reaction mixture with *A*. *terreus* GDH (*At*GDH); however, glucuronic acid was not detected. These results suggested that *Cp*GDH catalyzed the hydroxy group oxidation at the C6 position of glucose but not at the C1 position.

**Table 2:** Detection of gluconic acid and glucuronic acid contained in enzyme reaction mixture

Substrates	Concentration (mM)	
	Gluconic acid assay kit	Glucuronic acid assay kit
d-Glucose	ND^a^	ND
Oxidized product of d-Glucose (*At*GDH)	189	ND
Oxidized product of d-Glucose (*Cp*GDH)	ND	33.3

Glucose (50 mM) was used as a negative control. As *Colletotrichum plurivorum* glucose dehydrogenase (*Cp*GDH) produced little gluconic acid, a large amount of glucose oxidation product prepared with *Cp*GDH was necessary for detecting gluconic acid. Therefore, 500 mM glucose was oxidized with each GDH and used as the sample for evaluating gluconic acid. To detect glucuronic acid using the assay kit, 50 mM of glucose oxidation products prepared with each GDH were used.

a ND, not detected; *At*GDH, *Aspergillus terreus* glucose dehydrogenase.

### HPLC and MS analysis of the glucose oxidation product

**Figure 3:**
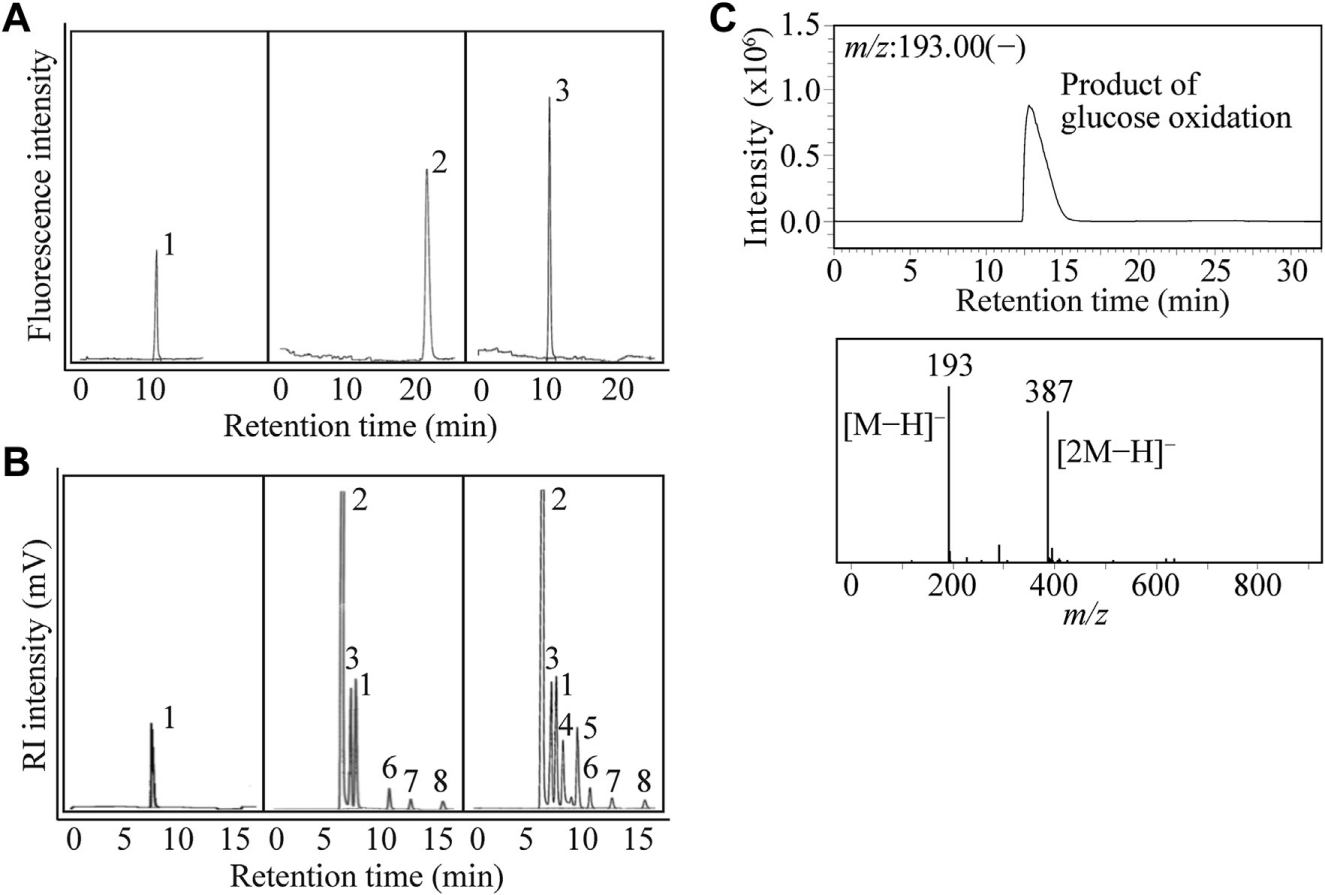
**HPLC chromatograms and MS chromatogram of the product obtained after glucose oxidation using *Colletotrichum plurivorum* glucose dehydrogenase (*Cp*GDH)**. *A*, HPLC chromatogram of *p*-aminobenzoic ethyl ester (ABEE)-labeled compounds. *Left* panel, glucuronic acid standard; center panel, d-glucose standard; *right* panel, the purified product of glucose oxidation with *Cp*GDH. *B*, HPLC chromatogram of unlabeled compounds obtained after glucose oxidation using *Cp*GDH. *Left* panel, glucuronic acid standard; *center* panel, unpurified product of glucose oxidation; *right* panel, unpurified product of glucose oxidation with authentic gluconic acid and glucuronolactone. Peak 1 in the *left panel*, authentic glucuronic acid; peak 1 in the center and *right panel*, the product of glucose oxidation; peak 2, 5-SA; peak 3, sodium phosphate buffer; peak 4, authentic gluconic acid; peak 5, authentic glucuronolactone; peaks 6 and 7, oxidized *tert*-butylhydroquinone (TBHQ); peak 8, TBHQ. *C*, MS chromatogram of the purified glucose oxidation product. The main peak corresponds to a molecular weight of 193, which is consistent with the molecular weight of glucuronic acid. 5-SA, 5-sulfosalicylic acid; MS, mass spectrometry.

The product of glucose oxidation prepared using *Cp*GDH was labeled with *p*-aminobenzoic ethyl ester (ABEE) at the reduced C1 site and analyzed using HPLC. The ABEE derivatives of authentic glucuronic acid and glucose were completely separated by HPLC with a fluorescence detector and detected at the retention times of 10 and 21 min, respectively (Fig. 3*A*). The ABEE derivative of the glucose oxidation product prepared with *Cp*GDH was detected at a retention time of 10 min, similar to that of the authentic glucuronic acid derivative; no other peaks were detected. The enzyme reaction mixture was also analyzed without ABEE derivatization by HPLC using a refractive index detector after deproteinization with 5-sulfosalicylic acid (5-SA). Peaks of the buffer, mediator, products of mediator oxidation, glucuronic acid, and residual 5-SA were detected (Fig. 3*B*). To confirm that the glucose oxidation product prepared with *Cp*GDH was not gluconic acid, authentic gluconic acid was added to the enzyme reaction mixture. Authentic glucuronolactone was also added to the enzyme reaction mixture as glucuronic acid, and glucuronolactone attain equilibrium state in solution depending on the pH and temperature. HPLC analysis revealed that peaks of the added authentic gluconic acid and glucuronolactone did not overlap with existing peaks of the enzyme reaction mixture (Fig. 3*B* right panel). These results demonstrated that *Cp*GDH specifically oxidized glucose to glucuronic acid, and other organic acids and lactones were not produced. Mass spectrometry (MS) analysis of the purified glucose oxidation product with *Cp*GDH revealed only one peak with the same molecular weight as that of glucuronic acid (*i*.*e*., 193) (Fig. 3*C*).

### NMR analysis of the glucose oxidation product

**Figure 4:**
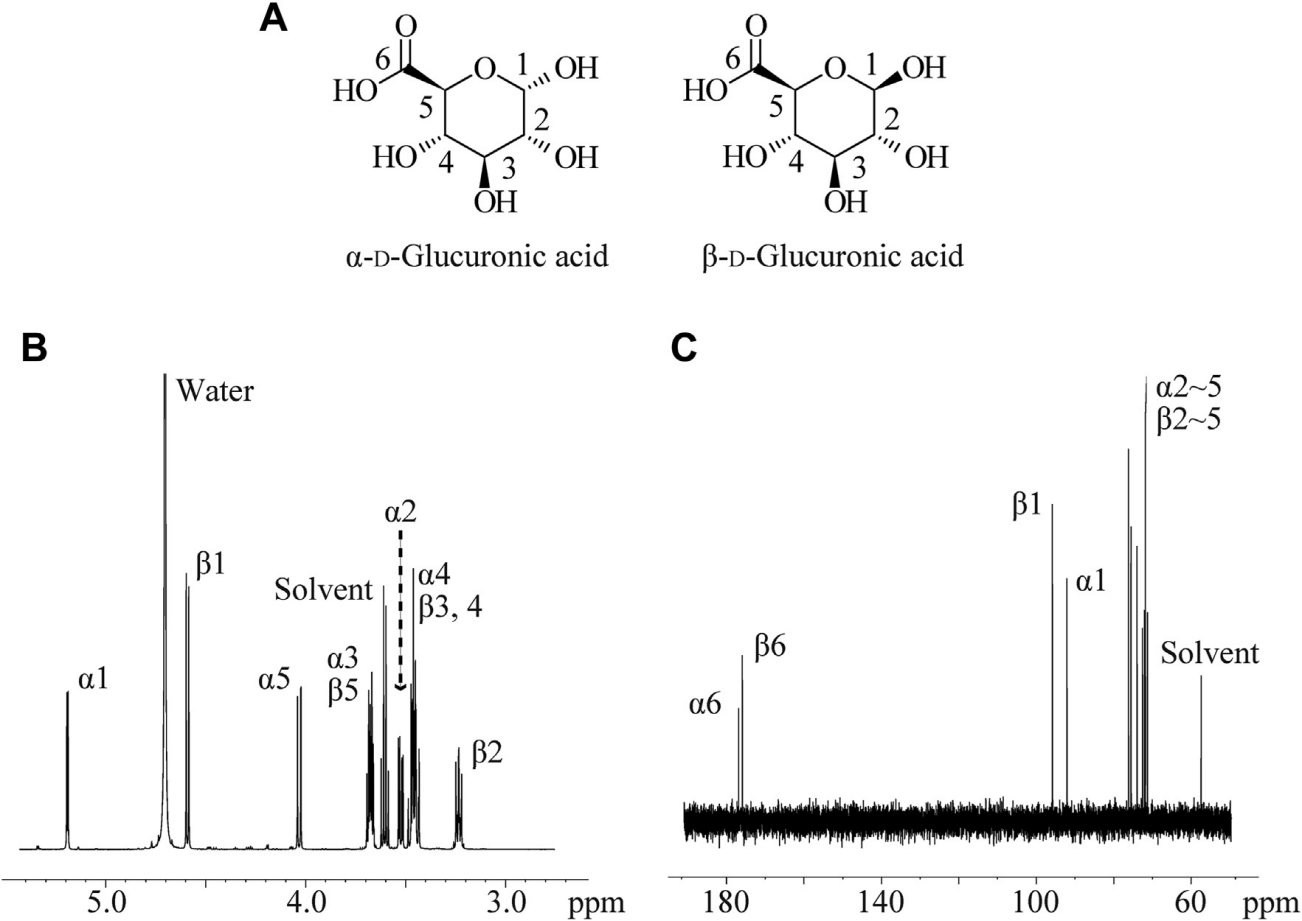
**Structure of glucuronic acid and the result of NMR**. *A*, structure of α-d-glucuronic acid and β-d-glucuronic acid. *B*, ^1^H-NMR spectra and (*C*) ^13^C-NMR spectra of the product of glucose oxidation using *Colletotrichum plurivorum* glucose dehydrogenase (*Cp*GDH). Ethanol was used as the solvent. The 600 MHz ^1^H NMR spectrum and 150 MHz ^13^C NMR spectrum of the product of glucose oxidation in D_2_O are shown below. ^1^H NMR (600 MHz, D_2_O): *δ* = 5.19 (*d*, *J* = 3.6 Hz, 1H), 4.59 (*d*, *J* = 8.4, 1H), 4.03 (*d*, *J* = 8.4 Hz, 1H), 3.66 to 3.69 (*m*, 2H), 3.52 (*dd*, *J* = 3.6 and 9.6 Hz, 2H), 3.43 to 3.49 (*m*, 3H), 3.24 (*dd*, *J* = 3.0 and 6.0 Hz, 1H). ^13^C NMR (150 MHz, D_2_O): δ = 176.7, 175.9, 95.9, 92.1, 76.2, 75.6, 74.0, 72.6, 72.1, 71.9, and 71.3.

Furthermore, we designed a one-pot glucose-oxidizing system and completely oxidized 1 M glucose using *Cp*GDH for 48 h. The glucose oxidation product was purified using a C18 column and further by ethanol precipitation to remove contaminants, such as the mediator, for NMR analysis. The results of ^1^H-NMR and ^13^C-NMR are shown in Fig. 4, *B* and *C*, respectively. The peaks found in ^1^H-NMR analysis (Fig. 4*B*) were attributed to the glucose oxidation product, water, and ethanol solvent. The peaks of hydrogen at the C1-position of α-d-glucuronic acid and β-d-glucuronic acid were found at 4.8 ppm and 5.2 ppm, respectively. This result demonstrated that the hydroxy group at the C1-position of glucose was not oxidized. The peaks found in ^13^C-NMR analysis (Fig. 4*C*) were attributed to the glucose oxidation product and ethanol solvent. The peaks around 180 ppm indicated that the C6-position of α-d-glucose and β-d-glucose were oxidized to carboxylic acid by *Cp*GDH. Overall, these results demonstrated that the glucose oxidation product obtained with *Cp*GDH is glucuronic acid and that *Cp*GDH is a novel G6DH.

### Stoichiometric ratio of the substrate and mediator

**Figure 5:**
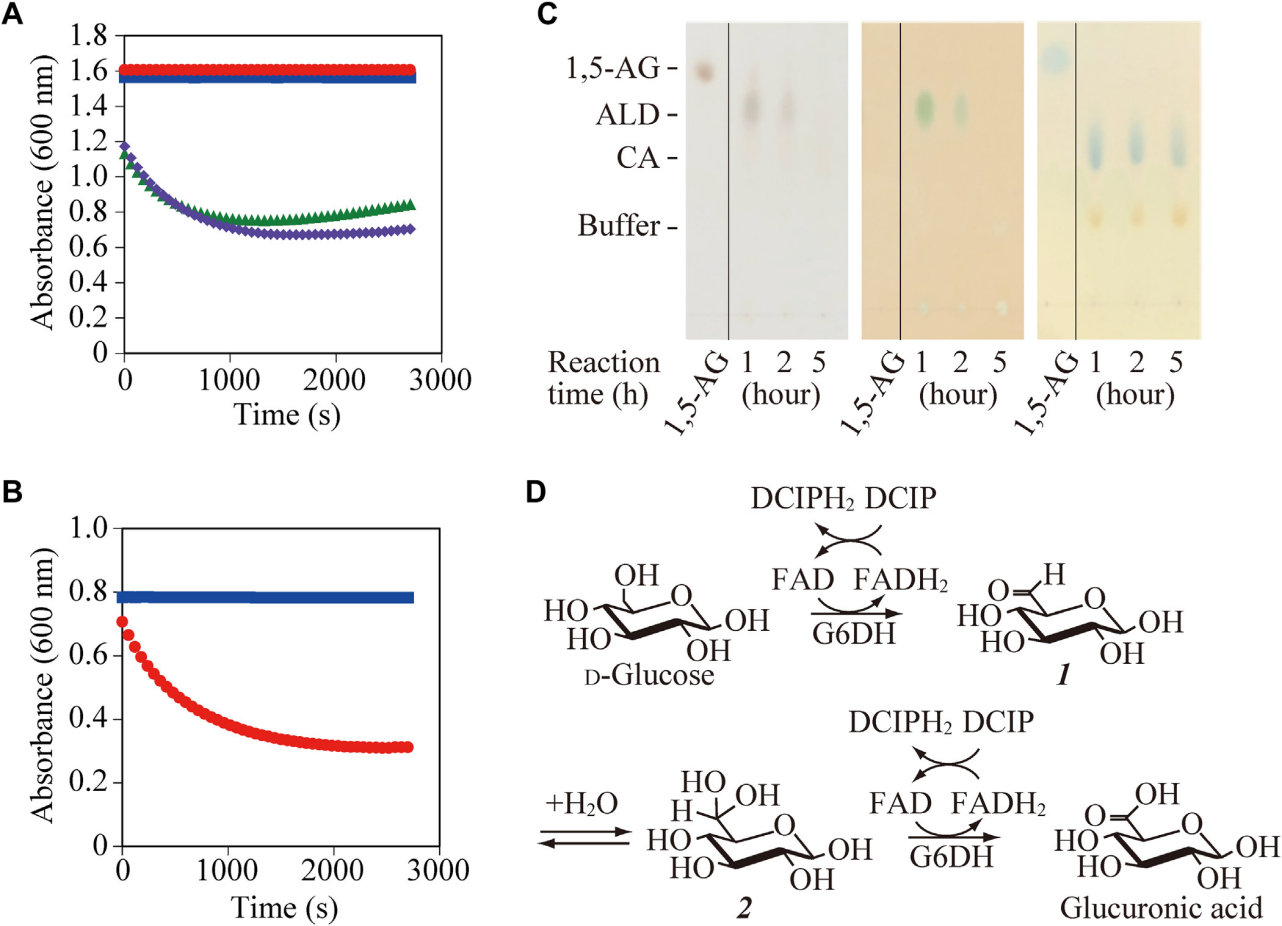
**Molar ratio of the substrate and mediator and the expected reaction process.***A*, to confirm the effect of dissolved oxygen on the absorbance behavior of the mediator 2,6-dichloroindophenol (DCIP), enzyme activity was evaluated using crude *Colletotrichum plurivorum* glucose dehydrogenase (*Cp*GDH) and a microplate spectrophotometer. Reactions were carried out with dissolved oxygen or without dissolved oxygen after its removal using nitrogen gas treatment. *Red circle*, without *Cp*GDH and dissolved oxygen; *blue square*, without *Cp*GDH and with dissolved oxygen; *green triangle*, with *Cp*GDH and dissolved oxygen; *purple diamond*, with *Cp*GDH and without dissolved oxygen. *B*, enzyme activity was assessed using purified *Cp*GDH and a spectrophotometer. The negative control (*blue*) lacked *Cp*GDH, whereas the *Cp*GDH activity assay (*red*) demonstrated the enzyme performance. Both assays used a reaction mixture purged of dissolved oxygen using nitrogen gas. *C*, TLC of the 1,5-AG oxidation product. Reaction mixtures collected at 1, 2, and 5 h after adding *Cp*GDH were developed on the TLC plate. Spots were visualized by spraying the plate with H_2_SO_4_ (*left*) or dipping in 2,4-dinitrophenylhydrazine (DNPH) for aldehyde detection (*center*) or bromocresol green (BCG) for acid detection (*right*). Each chromatogram had a splice between 1,5-AG and samples. *D*, expected reaction process of *Cp*GDH. First, β-d-glucose was oxidized to an aldehyde (1) by *Cp*GDH. Subsequently, a hydration reaction yielded an aldehyde hydrate, known as a gem-diol (2). Finally, *Cp*GDH oxidized gem-diol to a carboxylic acid, glucuronic acid. ALD, aldehyde; CA, carboxylate; Buffer, glycine-NaOH buffer (pH 10). 1,5-AG, 1,5-anhydro-d-glucitol .

Carboxylic acid is produced from primary alcohol *via* a two-step oxidation process of primary alcohol-to-aldehyde and aldehyde-to-carboxylic acid. If *Cp*GDH catalyzes both oxidation steps, two molecules of the mediator should be reduced for every molecule of glucose that is oxidized to glucuronic acid. We performed the enzyme activity assay using a reaction mixture containing excess amounts of the mediator 2,6-dichloroindophenol (DCIP) to determine the stoichiometric ratio of glucose to the mediator. The calibration curve obtained from oxidized DCIP at a known concentration (4.7–75 μM) was used to calculate the amount of reduced DCIP based on the oxidized DCIP absorbance value at the end of the enzyme reaction. For the experiment, we removed oxygen from a reaction mixture because the reduced DCIP was gradually reoxidized by dissolved oxygen. Microplate spectrophotometry (Fig. 5*A*) and spectrophotometry (Fig. 5*B*) were used for this assay. The decrease in absorbance stopped after 40 min of initiating the enzyme reaction, indicating that *Cp*GDH oxidized all of 12.5 μM glucose and reduced some of the excess DCIP. Based on this result and the value of oxidized DCIP absorbance, we determined that *Cp*GDH oxidized 12.5 μM glucose and reduced 23.4 μM DCIP. Hence, we concluded that the stoichiometric ratio of glucose to DCIP was 1:1.87 (approximately 1:2) and that *Cp*GDH catalyzed the two-step oxidation of glucose to glucuronic acid with the corresponding aldehyde as the intermediate.

### Detection of aldehyde intermediate

To demonstrate that aldehydes and carboxylic acids were produced from glucose by *Cp*GDH, an enzymatic reaction was carried out in a mixture containing 1,5-anhydro-d-glucitol (1,5-AG), glycine-NaOH buffer, *tert*-butylhydroquinone (TBHQ) as a mediator, and *Cp*GDH. The glucose analog 1,5-AG was the substrate and did not react with 2,4-dinitrophenylhydrazine (DNPH) as it is not aldose. Parts of the reaction mixtures collected at 1, 2, and 5 h were spotted onto different lanes of TLC plates. The separated spots were then stained with DNPH for aldehyde detection or bromocresol green (BCG) for acid detection (Fig. 5*C*). The 1, 5-AG spot disappeared after an hour (Fig. 5*C* left). The aldehyde spot was detected until 2 h after starting the enzymatic reaction and disappeared after 5 h (Fig. 5*C* center). Carboxylic acid was detected in all reaction mixtures (Fig. 5*C* right). These results indicated that *Cp*GDH catalyzes a two-step 1,5-AG oxidation from primary alcohol-to-aldehyde and aldehyde-to-carboxylic acid.

### The amino acid sequence of *Cp*GDH

**Figure 6:**
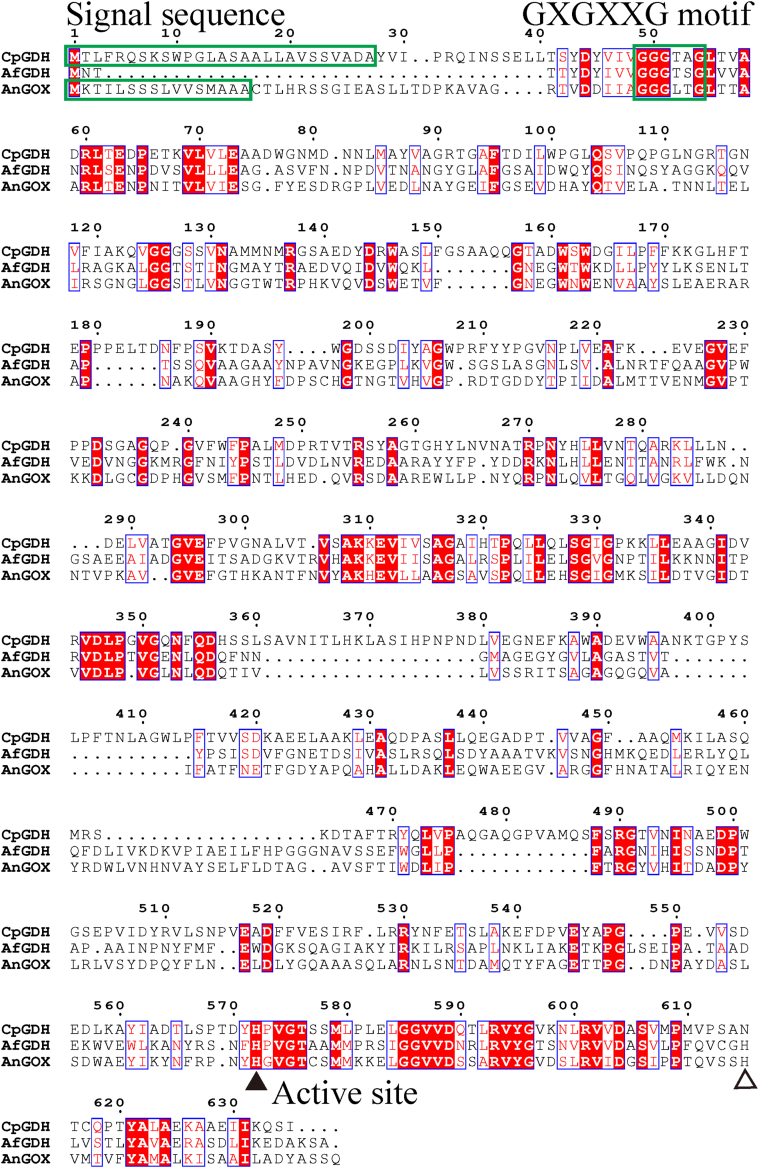
**The result of a multiple alignment using the amino acid sequences of *Colletotrichum plurivorum* glucose dehydrogenase (*Cp*GDH), *Aspergillus flavus* glucose dehydrogenase (*Af*GDH, PDB ID: 4YNU) and *Aspergillus niger* glucose oxidase (*An*GOX)**. Each signal sequence and the conserved GXGXXG motif known as FAD binding motif are surrounded with *green boxes*. The active site residues conserved in all enzymes are shown by a *black arrow*. Other active site residues conserved in *Af*GDH and *An*GOX are shown by a *white arrow*. PDB, Protein Data Bank

We analyzed the amino acid sequence of *Cp*GDH using Motif search and obtained the result showing that it is a type of glucose-methanol-choline oxidoreductase. The FAD-binding sequence motif of GXGXXG motif is detected in the amino acid sequence of *Cp*GDH. We evaluated the amino acid sequence homology of *Cp*GDH with other enzymes known as glucose-methanol-choline oxidoreductases including GDH from *Aspergillus flavus* (*Af*GDH, PDB ID: 4YNU) and glucose oxidase from *Aspergillus niger* (*An*GOX). The sequences of *Af*GDH and *An*GOX showed 28.9% and 29.0% homology to that of *Cp*GDH, respectively. A multiple alignment of the aforementioned three enzymes was conducted, using MAFFT (19). The result was analyzed by ESPript3.0 (Fig. 6). Different secretion signal sequences were detected in *Cp*GDH and *An*GOX at their N terminus. In all sequences, the GXGXXG motif and the histidine residue (black triangle) are conserved. Since the conserved histidine residues in *Af*GDH and *An*GOX were reported to form active site, we concluded that the His572 indicated by black arrow is an active amino acid residue of *Cp*GDH (20, 21). Although *Af*GDH and *An*GOX have another conserved histidine residue indicated by white arrow, asparagine residue was found in *Cp*GDH. Using the amino sequence of *Cp*GDH as a query sequence, we performed BLAST search to investigate the proteins with high sequence homology. Some sequences annotated as choline dehydrogenases and dehydrogenase *xptC* with more than 80% homology were found, while their functions were not characterized enough to make detail discussion.

### Analysis of the three-dimensional structure model of *Cp*GDH

**Figure 7:**
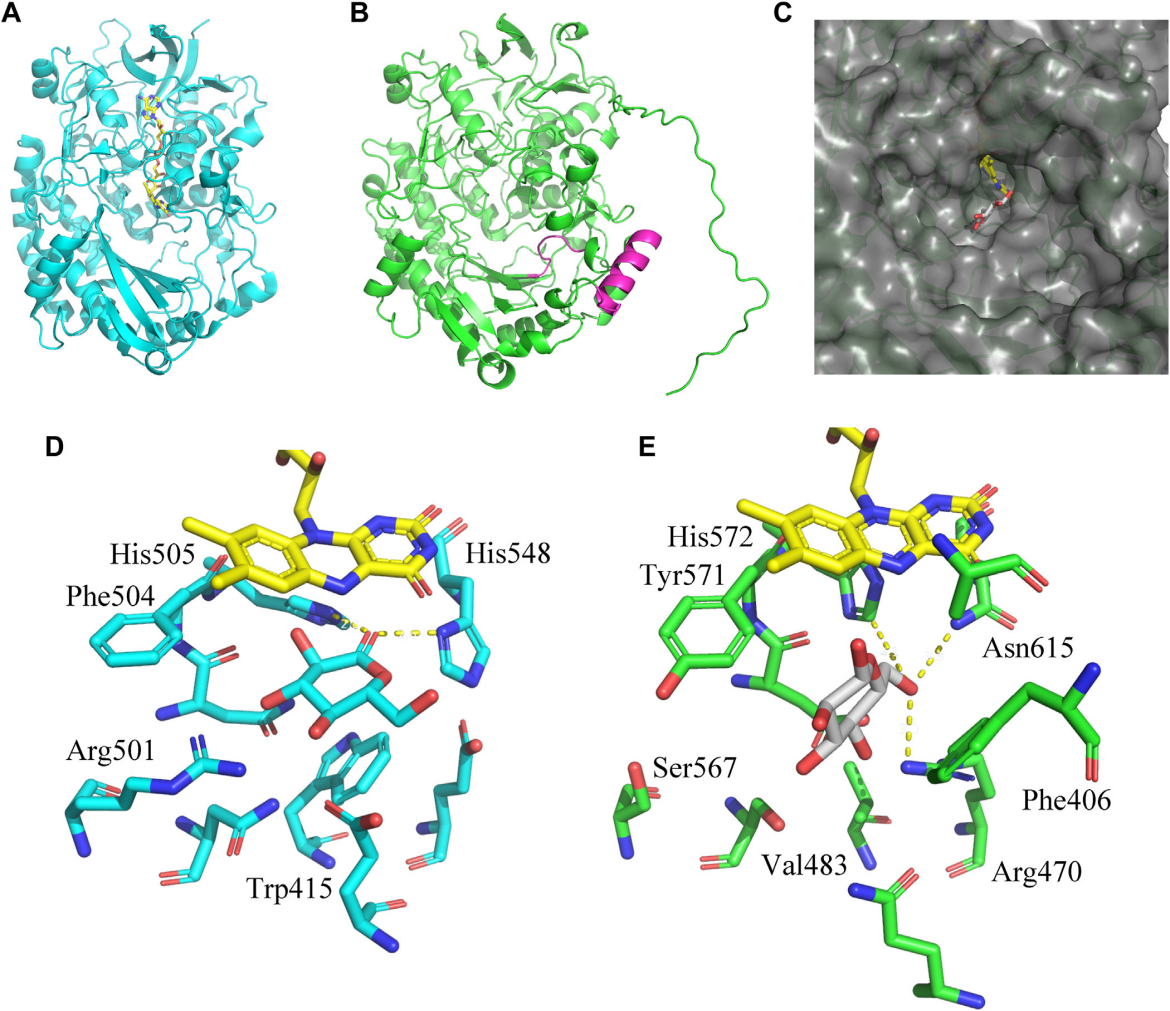
**The 3D structures of *Aspergillus flavus* glucose dehydrogenase (*Af*GDH, PDB ID: 4YNU) and *Colletotrichum plurivorum* glucose dehydrogenase (*Cp*GDH), and comparison of the amino acid residues at their substrate-binding pocket**. The 3D structures of 4YNU (*A*) and *Cp*GDH (*B*) are shown. *C*, enlarged view of the substrate-binding pocket of *Cp*GDH with an FAD and a substrate glucose. The 3D structures of *Af*GDH with d-glucono-1,5-lactone (*D*) and *Cp*GDH with glucose (*E*) are shown. A cofactor FAD and glucose are shown by *yellow stick* and *gray stick*. PDB, Protein Data Bank.

We created a *Cp*GDH structure model using SWISS-MODEL and compared it with that of 4YNU (Fig. 7, *A* and *B*). Their 3D structures are very similar, while they showed 28.9% amino acid sequence homology. *Cp*GDH has the unique α-helix and free loop shown in magenta. In particular, the free loop protruding into the substrate-binding pocket is important for the substrate specificity because Phe406 that sandwiches the substrate glucose is located on it. Unlike 4YNU, *Cp*GDH has the widely opened substrate-binding pocket shown in Fig. 7*C*. To investigate the oxidation mechanism of glucose-C6 by *Cp*GDH, we performed docking simulation of *Cp*GDH with a substrate β-d-glucose, using AutoDock Vina (https://vina.scripps.edu/) (22). On the other hand, we did not perform a docking simulation between 4YNU and the substrate, glucose. The Protein Data Bank (PDB) data 4YNU are registered as a cocrystal of *Af*GDH and D-glucono-1,5-lactone (LGC). Since LGC was used to obtain the ligand-complexed structure of *Af*GDH, we believed that we can speculate how *Af*GDH is bound to the substrates from 4YNU. The structures around a substrate-binding pocket of 4YNU and *Cp*GDH are shown in Fig. 7, *D* and *E*. The active site residues of 4YNU are His505 and His548 (21). The substrate glucose is bound to *Cp*GDH in a unique manner. Although the position-C1 of LGC is located near the active site residues His572 and His548 in *Af*GDH that catalyzes the oxidation of the portion-C1 of glucose, the position-C6 of glucose is located near the active site residue His572 in *Cp*GDH. The amino acid residues Arg470 and Asn615 are also located near the portion-C6 of glucose in *Cp*GDH. The amino acid residues Trp415 and Arg501 are important for the binding of *Af*GDH and LGC in the state shown in 4YNU. If they were changed to other amino acids, *Af*GDH and LGC would not be able to bind in the manner shown by 4YNU. However, the amino acid residues corresponding to Trp415 and Arg501 in 4YNU are changed to Val483 and Ser567 in *Cp*GDH, which significantly change the glucose-binding manner within *Cp*GDH. Moreover, in *Cp*GDH, glucose is sandwiched by Phe406 and Tyr571 and binds to *Cp*GDH in a tilted state relative to the isoalloxazine ring of FAD.

### Phylogenetic tree of the glucose-methanol-choline oxidoreductase family

**Figure 8:**
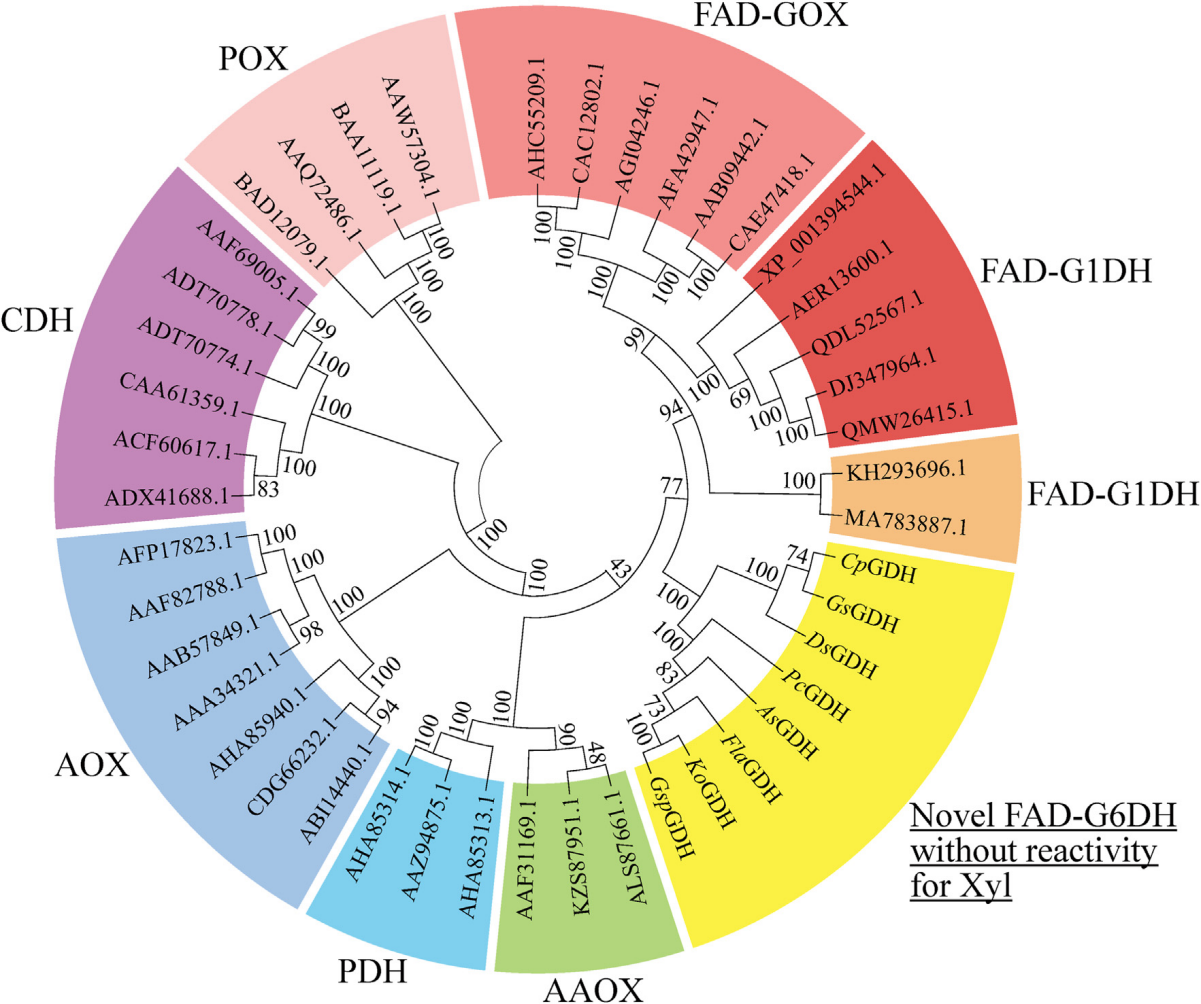
**Phylogenetic tree of FAD-dependent oxidoreductases**. The phylogenetic tree was created using the amino acid sequences of previously characterized FAD-dependent oxidoreductases. They were obtained from CAZy, a database of sugar-related enzymes, patents, and treatises. The amino acid sequences of enzymes in the phylogenetic tree are those from AHC55209.1 (*Aspergillus carbonarius*), CAC12802.1 (*Aspergillus niger*), AGI04246.1 (*Aspergillus niger*), AFA42947.1 (*Penicillium chrysogenum*), AAB09442.1 (*Talaromyces flavus*), CAE47418.1 (*Talaromyces variabilis*), XP_001394544.1 (*Aspergillus niger*), AER13600.1 (*Colletotrichum gloeosporioides*), QDL52567.1 (*Aspergillus terreus*), DJ347964.1 (*Aspergillus oryzae*), QMW26415.1 (*Aspergillus flavus*), KH293696.1 (*Mucor prainii*), MA783887.1 (*Mucor hiemalis*), LC739014 (*Cp*GDH, *Colletotrichum plurivorum*), LC739015 (*Gs*GDH, *Glomerella* sp), LC739018 (*Ds*GDH, *Dioszegia* sp.), LC739019 (*Pc*GDH, *Phialemoniopsis curvata*), LC739017 (*As*GDH, *Acremonium strictum*), LC739016 (*Fla*GDH, *Fusarium langsethiae*), LC739020 (*Ko*GDH, *Khuskia oryzae*), LC739021 (*Gsp*GDH, *Gaeumannomyces* sp.), ALS87661.1 (*Pycnoporus cinnabarinus*), KZS87951.1 (*Sistotrema strumniveocremeum*), AAF31169.1 (*Pleurotus pulmonarius*), AHA85313.1 (*Agaricus campestris*), AAZ94875.1 (*Leucoagaricus meleagris*), AHA85314.1 (*Agaricus xanthodermus*), ABI14440.1 (*Gloeophyllum trabeum*), CDG66232.1 (*Phanerodontia chrysosporium*), AHA85940.1 (*Taiwanofungus camphoratus*), AAA34321.1 (*Candida boidinii*), AAB57849.1 (*Komagataella pastoris*), AAF82788.1 (*Passalora fulva*), AFP17823.1 (*Aspergillus terreus*), ADX41688.1 (*Trametes cinnabarina*), ACF60617.1 (*Gelatoporia subvermispora*), CAA61359.1 (*Phanerodontia chrysosporium*), ADT70774.1 (*Dichomera saubinetii*), ADT70778.1 (*Stachybotrys bisbyi*), AAF69005.1 (*Humicola insolens*), BAD12079.1 (*Lyophyllum shimeji*), AAQ72486.1 (*Phlebiopsis gigantea*), BAA11119.1 (*Trametes versicolor*), and AAW57304.1 (*Trametes pubescens*).

We performed a BLAST search using the *Cp*GDH amino acid sequence as a query to find G6DH candidate genes. Genes with sequence homology were amplified using genomic DNA extracted from the corresponding microorganisms as templates. *A*. *oryzae* was transformed with G6DH candidate genes. Overall, 12 G6DH candidates expressed were purified using column chromatography, and their activities in oxidizing the hydroxy group at the C6 position of glucose were confirmed (Fig. S1). Their enzymatic properties are listed in Table S2. To characterize the carbohydrate-related enzyme group consisting of G6DHs, we constructed a phylogenetic tree using the amino acid sequences of carbohydrate-related enzymes selected from the literature and the AA3 family in the CAZy database (Fig. 8). Our results indicate that G6DHs are not classified into any known group but form a new group of oxidoreductases.

### Enzyme activity and kinetic parameters of *Cp*GDH

The kinetic parameters of *Cp*GDH and the widely known *At*GDH are presented in Table 3. The *k*_cat_ and *k*_cat_/*K*_m_ values of *Cp*GDH for glucose were 12-fold and 73-fold lower than that of *At*GDH. As *Cp*GDH also showed high enzyme activities in the reaction mixture including 1,5-AG or 2-deoxy-d-glucose, we evaluated the kinetic parameters using those substrates. The *k*_cat_ values for glucose and 1,5-AG are similar. The *k*_cat_ value for 2-deoxy-d-glucose is 2-fold higher than that of glucose. The *k*_cat_/*K*_m_ value for glucose is 2.7-fold lower than that for 1,5-AG and 1.9-fold higher than that for 2-deoxy-d-glucose. No enzymatic activity was detected in the oxidase activity assay using 4-aminoantipyrine, indicating that *Cp*GDH was a dehydrogenase that could not use molecular oxygen as an electron acceptor. For use as a glucose sensor, the substrate specificity of *Cp*GDH was superior to that of *At*GDH; however, the kinetic parameters of *Cp*GDH were inferior to those of *At*GDH.

**Table 3:** Activities and kinetic parameters of *Aspergillus terreus* glucose dehydrogenase (*At*GDH) and *Colletotrichum plurivorum* GDH (*Cp*GDH)

GDHs	Substrate	Specific activity	Oxidase activity	*K*_m_	*k*_cat_	*k*_cat_/*K*_m_
		(U/mg)	(U/mg)	(mM)	(s^−1^)	(s^−1^ mM^−1^)
*At*GDH	Glc	2680 ± 20.9	ND^a^	68.2 ± 0.50	3550 ± 16.7	52.1
*Cp*GDH	Glc	119 ± 1.29	ND	398 ± 7.21	285 ± 2.96	0.72
	1,5-AG	170 ± 3.63	-	131 ± 10.8	256 ± 5.80	1.95
	2dGlc	91.7 ± 0.94	-	1420 ± 60.6	540 ± 17.4	0.38

One unit is defined as the amount of enzyme that converts 1 μmole of glucose to gluconic acid or glucuronic acid per minute. Specific activities of dehydrogenase and oxidase toward glucose were determined using a 2,6-dichloroindophenol assay or 4-aminoantipyrine assay. Glc, d-glucose; 1,5-AG, 1,5-anhydro-d-glucitol; 2dGlc, 2-deoxy-d-glucose.

a ND, not detected.

### Properties of *Cp*GDH

The properties of *Cp*GDH are shown in Fig. S2. The absorbance maxima of purified *Cp*GDH were observed at 266, 388, and 460 nm and that of *At*GDH were observed at 275, 381, and 460 nm (Fig. S2*A*). The typical flavoprotein spectrum shows the almost same characteristics (23, 24). To evaluate the number of FAD binding to a molecule of *Cp*GDH, AMP assay was performed. Neither treatment of the enzyme heated at 95 °C for 10 min nor addition of Na_2_S_2_O_4_ to the enzyme solution showed any AMP release. These results suggested the presence of covalently bound flavin in the enzyme. On the other hand, approximately 1 mol of AMP per mol of *Cp*GDH was detected after trypsin digestion followed by phosphodiesterase treatment. These data indicated that *Cp*GDH contains covalently bound flavin compound, probably FAD, at 1 mol per mol of enzyme. Therefore, these results demonstrated that *Cp*GDH is a flavoprotein with an FAD. The enzyme activity of *Cp*GDH was maintained in various buffers (pH 4–10) and was hardly inactivated by incubation from 4 °C to 40 °C (Fig. 2, *B* and *C*). *Cp*GDH was inhibited by the heavy metal salt copper (II) dichloride but not by the surfactant Triton X-100, hydrogen peroxide, and metal salt iron (III) trichloride (Fig. S2*D*). Furthermore, we evaluated the substrate specificity of *Cp*GDH toward sugars and their analogs (Table S3). *Cp*GDH also showed high reactivity toward the nonreducing saccharides 1,5-AG and 2-deoxy-d-glucose. In contrast, *Cp*GDH did not oxidize xylose and glucose 6-phosphate. The mediators reduced by *Cp*GDH are shown in Table S4. The positively charged compounds, except thionine, ascorbic acid, and cytochrome c, functioned as mediators, whereas the negatively charged compounds did not.

## Discussion

Many types of cofactor-dependent GDHs, such as FAD-GDH, NAD-GDH, and pyrroloquinoline quinone-dependent GDH, have been reported. *At*GDH, with superior substrate specificity, is a FAD-GDH important for human health because it is present in SMBGs. However, a novel GDH without xylose reactivity is required for developing SMBG devices with a more sophisticated sensor.

We thus screened various microorganisms and isolated a novel FAD-dependent G6DH without xylose reactivity from *C*. *plurivorum*. To date, only one study has reported a saccharide oxidase catalyzing hydroxy group oxidation at the C6 position of glucose, and this was a mutated galactose oxidase with very low enzymatic activity (25). We characterized CpGDH using recombinant CpGDH expressed from an *A*. *oryzae* transformant and demonstrated that it oxidized glucose at the C6-position but not maltose or xylose.

*Cp*GDH and *At*GDH show almost same spectra that are same as those of other flavoproteins, indicating that *Cp*GDH is an FAD-dependent oxidoreductase. *Cp*GDH is stable with respect to pH and temperature. *Cp*GDH showed high reactivity for glucose, 1,5-AG, and 2-deoxy-d-glucose but hardly showed any reactivity for galactose, xylose, and trehalose. The *k*_cat_ values for 1,5-AG and glucose are similar, but *K*_m_ value for 1,5-AG is lower than that of glucose. The hydrophobic amino acid residues that are located around the C1 position of glucose might allow 1,5-AG with less hydroxy groups than glucose to stably bind to *Cp*GDH (Fig. 7*A*). We further concluded that the hydroxy group at the C4 position of hexose could be important for substrate recognition because galactose was not oxidized by *Cp*GDH. Cellobiose components, found in cell walls, and trehalose sweeteners are not present in the human blood. Further, 1,5-AG, a biomarker of hyperglycemia that reflects the average blood glucose levels during the previous 2 weeks, is present in human blood at only one-tenthth of the amount of glucose. Hence, *Cp*GDH without reactivity to maltose and xylose can accurately evaluate blood glucose levels and is a useful oxidoreductase for developing SMBG devices. The results of TLC, HPLC, and NMR analysis demonstrated that the glucose oxidation product of *Cp*GDH was glucuronic acid and that no other products of glucose oxidation were detected. This result demonstrated that *Cp*GDH is a novel G6DH that specifically catalyzes hydroxy group oxidation at the C6 position of glucose.

To determine the reaction scheme of glucose oxidation by *Cp*GDH, we calculated the stoichiometric ratio of glucose to DCIP based on the results of the activity assay and demonstrated that *Cp*GDH reduced two molecules of DCIP when one molecule of glucose was oxidized. This finding indicated that *Cp*GDH catalyzed both reactions in the two-step oxidation of primary alcohol-to-aldehyde and aldehyde-to-carboxylic acid. This reaction scheme is similar to that of FAD-dependent aryl alcohol oxidases (AAOXs). AAOXs catalyze the oxidation of primary alcohols to carboxylic acids; the produced aldehyde is then oxidized to carboxylic acid *via* a gem-diol, which is formed by aldehyde hydration (26). Enzymatic two-step oxidation reaction was also reported in some other articles (27, 28). Therefore, we considered that the reaction scheme of *Cp*GDH was similar to that of AAOX, as shown in Fig. 5*D*. First, *Cp*GDH oxidizes one molecule of glucose and reduces one molecule of DCIP. Next, the aldehyde produced by glucose oxidation is hydrated with water to form a gem-diol. Finally, *Cp*GDH oxidizes the gem-diol to a carboxylic acid and reduces another one molecule of DCIP. However, we considered that *Cp*GDH was a different enzyme from AAOX. Notably, *Cp*GDH did not oxidize alcohols, such as benzyl alcohol, cinnamyl alcohol, and veratryl alcohol, which have been used to assess the substrate specificity of AAOX (29, 30). The sequence homology between the amino acid sequence of *Cp*GDH to those of *Af*GDH or *An*GOX known as FAD-dependent oxidoreductases was under 30%. The amino acid residues in the substrate-binding pocket of *Af*GDH and *Cp*GDH were different significantly. For instance, the hydrophobic amino acid residue Trp415 is located to lift LGC toward FAD in *Af*GDH, but that of Phe406 is positioned to support glucose toward FAD in *Cp*GDH. The amino acid corresponding to Trp415 in *Af*GDH is a small Val483 in *Cp*GDH, indicating that the spatial allowance allows glucose to bind in a tilted state and *Cp*GDH has the substrate binding manner different from *Af*GDH. Even in the phylogenetic tree generated from amino acid sequences encoding FAD-dependent oxidoreductases, G6DHs formed a different group from that of G1DHs and AAOXs. FAD-GDHs (KH293696.1 and MA783887.1) derived from the genus *Mucor* show little reactivity to xylose and are classified into a group similar to known G1DH and glucose 1-oxidase. Further, 3D-structural models of *Cp*GDH and FAD-GDHs (KH293696.1 and MA783887.1, respectively) prepared using SWISS-MODEL were compared. The amino acid placement of KH293696.1 and MA783887.1 was similar but significantly different from that of *Cp*GDH. Therefore, we concluded that KH293696.1 and MA783887.1 are G1DHs, and *Cp*GDH is different from the known FAD-dependent oxidoreductases.

The discovery of G6DHs is important for creating more accurate glucose sensors. Notably, it is also important for preventing plant pathogenic fungi, which is necessary to improve the grain productivity per farmland to support the increasing world population. Plants accumulate many types of glycosides, and some aglycones show antibacterial activity. When pathogens infect plants or break their tissues, antibacterial agents are produced from plant glucosides by pathogenic glycosidases, which suppress pathogen infection (31, 32). We suspected that *Cp*GDH, which exerts cellobiose oxidation activity, can also oxidize glycosides; this is because glycosides are substances in which one of the sugars constituting cellobiose is replaced with another functional group. If G6DHs oxidize glycosides, the oxidized products are not degraded to aglycones by glucosidases, and plants cannot exclude pathogens. Most G6DHs have been isolated from phytopathogenic filamentous fungi, suggesting that G6DHs may be involved in evading plant immunity. Therefore, we believe that knowledge of G6DHs will contribute to the development of new pesticides with selective inhibitory activity against pathogenic fungal infections. Based on this hypothesis, we plan to focus on polyphenol aglycones with various physiological activities and assess the reactivity of *Cp*GDH to polyphenol glucosides.

Thus, G6DHs are fascinating oxidoreductases from both academic and industrial aspects. We thus expect that these enzymes will contribute to the development of the medical, agricultural, and research fields.

## Experimental procedures

### Cloning of the cDNA encoding *Cp*GDH

*C*. *plurivorum* was grown in a culture medium containing 2% (w/v) dextrin (FUJIFILM Wako Pure Chemical), 1% (w/v) polypeptone (FUJIFILM Wako Pure Chemical), 0.5% (w/v) potassium dihydrogen phosphate (NACALAI TESQUE), and 0.05% (w/v) magnesium sulfate heptahydrate (NACALAI TESQUE). After 3 days of cultivation at 25 °C, fungal cells were collected and frozen at −80 °C until further analysis. Next, total RNA was extracted using ISOGENII (Nippon Gene). A complementary DNA (cDNA)) library was prepared from the total RNA using the SMARTer rapid amplification of cDNA ends (RACE) cDNA Amplification Kit (Takara Bio), according to the manufacturer’s instructions. Oligonucleotide primers, F1: CGCAGCTCGTCAAAATGACGCTCTTTCGCCAGTC and R1: GTTCATTTAGATGCTCTGCTTGATGAT, were used to amplify the *cp*gdh gene fragment and were designed based on *gdh* sequences registered in GenBank and those that we have collected so far. Furthermore, the full-length nucleotide sequence of *cp*gdh was obtained using 5′ rapid amplification of cDNA ends (RACE) and 3′ RACE. The *cp*gdh gene was amplified using PCR, and the expression plasmid was constructed based on previous studies (33, 34, 35). The plasmid was then transformed into *Escherichia coli* JM109 (Takara Bio) using heat shock, according to the manufacturer’s instructions. After 16 h of plate inoculation, a single colony of the transformed *E*. *coli* JM109 was collected and cultured in 10 ml of LB for another 16 h at 37 °C. The plasmid was extracted using the QIAprep Spin Miniprep Kit (Qiagen), and the nucleotide sequences of the inserted genes were analyzed (36). The extracted plasmid was used for the transformation of *A*. *oryzae*; the transformants were then used for protein expression. The cells were cultured on Czapek Dox Agar, and transformants carrying the plasmid were isolated. Moreover, the genes coding G6DH except for *Cp*GDH were isolated from different microorganisms using a BLAST search based on the *cp*gdh sequence, and the plasmid that incorporated these genes was used for *A*. *oryzae* transformation.

### Production and purification of *Cp*GDH

For protein expression, the selected colony was inoculated into 10 ml of a liquid medium comprising 2% (w/v) dextrin (FUJIFILM Wako Pure Chemical), 1% (w/v) polypeptone (FUJIFILM Wako Pure Chemical), potassium dihydrogen phosphate (NACALAI TESQUE), and 0.05% (w/v) magnesium sulfate heptahydrate (NACALAI TESQUE). After cultivation for 120 h at 30 °C, the media containing *Cp*GDH were collected to determine enzyme activity. Further, 150 ml of liquid medium was used to produce a large amount of *Cp*GDH and was purified. Ammonium sulfate (FUJIFILM Wako Pure Chemical) was added to the culture supernatant, the unnecessary protein was precipitated, and the supernatant containing *Cp*GDH was collected. The supernatant was then applied to a Butyl 650-C column (Tosoh) preequilibrated with an equilibration buffer composed of 50 mM potassium phosphate buffer (pH 7.5) and 55% ammonium sulfate. After applying the supernatant, the column was washed with the equilibration buffer. The target protein was then eluted with 50 mM potassium phosphate buffer (pH 7.0) containing 20% ammonium sulfate, and the purified fraction was demineralized. The supernatant was applied to a DEAE A-500m column (JNC), which was preequilibrated with 120 mM potassium phosphate buffer (pH 6.0) and then washed with the same buffer. The target protein was eluted with 150 mM potassium phosphate buffer (pH 6.0). The purified protein (0.5 mg/ml) was then mixed with 100 mM potassium phosphate buffer (pH 6.0) and 5 U/ml endoglycosidase (Roche), followed by incubation at 25 °C to remove glycans. Subsequently, aliquots of the purified protein were diluted with distilled water and mixed with 2 × loading buffer and β-mercaptoethanol (FUJIFILM Wako Pure Chemical), which is a reducing agent. The resulting mixture was boiled at 100 °C for 3 min. Boiled specimens and protein size markers (SMOBIO Technology) were then loaded onto 12.5% polyacrylamide gels (ATTO). SDS-PAGE was performed at 20 mA, and the proteins were stained with Coomassie blue. The eluted fractions were subjected to kinetic assays, substrate specificity assays, and the preparation of substrate oxidation products. Other G6DHs except for *Cp*GDH were purified only by DEAE column. Briefly, the supernatant was applied to a DEAE 650-S column (Tosoh), which was preequilibrated with 50 mM potassium phosphate buffer (pH 6.0) and then washed with 120 mM potassium phosphate buffer (pH 6.0). The target proteins were eluted with 200 mM potassium phosphate buffer (pH 6.0).

### GDH activity assay

A reaction mixture comprising 333 mM glucose (NACALAI TESQUE), 33 mM potassium phosphate buffer (pH 6.0), 0.2 mM 1-m-PMS (TCI), and 0.14 mM DCIP (Merck) was prepared. The enzyme activity and specific activity of GDHs were calculated using an absorption coefficient of 10.8 mM^−1^ cm^−1^ at 600 nm (37). For enzyme screening, we used 50 mM glucose, maltose, xylose, and galactose to evaluate substrate specificity. To obtain kinetic parameters, enzyme activities were determined using the same procedure, wherein substrate solutions of various concentrations were added. To determine the substrate-specific activity of purified *Cp*GDH, a reaction mixture containing 100 mM substrate or 50 mM 1,5-AG was used. A spectrophotometer V-660 (JASCO) was used to evaluate the kinetic parameters of *Cp*GDH, specific activity of *Cp*GDH, and reaction ratio of the substrate to the mediator. A SpectraMax PLUS 384 microplate spectrophotometer (Molecular Devices) was used for other assays. All substrates, except glucose, were purchased from FUJIFILM Wako Pure Chemical, NACALAI TESQUE and TCI.

### Protein assay

Protein concentrations were evaluated using Bradford reagent (Bio-Rad) in accordance with the instruction manual. Briefly, the Bradford reagent was diluted 5-fold in water to prepare a working reagent. The working reagent was then mixed with enzyme solutions, and the absorbance was measured at 595 nm using a microplate spectrophotometer.

### Glucose oxidase activity assay

Oxidase activity was determined by measuring the amount of hydrogen peroxide produced during substrate oxidation using an absorption coefficient of 5.3 mM^−1^ cm^−1^ at 500 nm (38). The glucose oxidation activity was measured using a microplate spectrophotometer. The reaction mixture contained 33 mM potassium phosphate buffer (pH 7.0), 0.8 mM 4-aminoantipyrine (FUJIFILM Wako Pure Chemical), 14 mM phenol (FUJIFILM Wako Pure Chemical), 3.3 U/ml horseradish peroxidase (FUJIFILM Wako Pure Chemical), and 333 mM glucose.

### Cofactor characterization

To confirm the FAD spectra of GDHs, 12 μM purified *Cp*GDH and 19 μM purified *At*GDH were used as specimens. FAD absorption spectra were recorded using a spectrophotometer V-660 at 25 °C. Glucose (50 mM) was added as a substrate to the purified GDHs to record the reduced FAD spectrum.

### Molecular mass determination with gel filtration analysis

Purified *Cp*GDH (0.4 mg) was applied onto Superdex 200 INCREASE 10/300 GL column (GE HealthCare) using the ÄKTA FPLC system (GE HealthCare). The column was preequilibrated with 20 mM Tris–HCl (pH 7.5) containing 100 mM NaCl. The enzyme was isocratically eluted at a flow rate of 0.35 ml/min monitoring UV absorbance at 280 nm. Standard protein mixture (Bio-Rad Laboratories, Inc) and blue dextran were used to draw a standard curve for molecular mass determination.

### Identification of FAD by liberation of AMP

Purified recombinant *Cp*GDH protein was subjected to identify the prosthetic group with the methods described previously (39) with slight modification. Briefly, purified *Cp*GDH solution was either heated at 95 °C for 10 min or added with Na_2_S_2_O_4_. The heated sample was farther digested with trypsin followed by phosphodiesterase treatment. Treated samples were analyzed to detect AMP by HPLC equipped with Cosmosil 5C_18_-AR-II column (NACALAI TESQUE, 4.6 × 150 mm), isocratically eluted with the buffer solution (200 mM KH_2_PO_4_:methanol = 9:1), monitoring UV absorbance at 254 nm.

### pH stability assay

To determine the influence of pH on *Cp*GDH stability, an enzyme solution of 6 U/ml *Cp*GDH was mixed with each 100 mM buffer, including sodium citrate buffer (pH 2.2–7.0), potassium acetate buffer (pH 3.0–6.0), potassium phosphate buffer (pH 6.0–8.0), Tris–HCl buffer (pH 7.0–9.0), and glycine-NaOH buffer (pH 9.0–10.0). These solutions were incubated at 30 °C for 60 min, and residual activity was determined using an activity assay with DCIP, similar to the GDH activity assay. Next, the activity of incubated *Cp*GDH was compared with that of unincubated *Cp*GDH, and the ratio of residual activity was calculated.

### Thermal stability assay

The enzyme solution containing 6 U/ml *Cp*GDH and 100 mM potassium phosphate buffer (pH 8.0) was incubated for 60 min at various temperatures, and the activity of the solution was determined using DCIP. To obtain the ratio of residual activity, the activity of incubated *Cp*GDH was compared with that of incubated *Cp*GDH at 4 °C.

### Enzyme inhibitory assay

The inhibitory effects of some materials, known as GDH inhibitors, were determined to measure the *Cp*GDH activity. The assay was performed in a reaction mixture containing 33 mM potassium phosphate buffer (pH 7.0), 333 mM glucose, 0.14 mM DCIP, 0.20 mM 1-m-PMS, 0.06 U/ml *Cp*GDH, and some inhibitor candidates, such as hydrogen peroxide, Triton X-100, FeCl_3_, and CuCl_2_. Subsequently, the results were compared with the values measured in the absence of inhibitors, and the ratio of residual activity was calculated.

### Preparation of the glucose oxidation product

To determine whether the product of glucose oxidation was glucuronic acid, 1-m-PMS or TBHQ (TCI) was used to prepare the product of glucose oxidation by *Cp*GDH. The color of the reaction mixture, which contained 1-m-PMS or TBHQ, changed when substrate oxidation was complete; therefore, we could determine whether the enzyme reaction was complete. Briefly, the reaction mixture with 10 mM 1-m-PMS comprised 100 mM buffer (pH 7.0), 50 mM glucose, 500 U/ml catalase, and 20 to 40 U/ml *Cp*GDH. The reaction mixture with 10 mM TBHQ comprised 100 mM sodium phosphate buffer (pH 8.0), 50 mM glucose, 500 U/ml catalase, 0.5 U/ml laccase (Merck) as a TBHQ oxidase, and 20 to 40 U/ml *Cp*GDH. The color of the reaction mixture, including guaiacol (TCI), remained almost unchanged, whereas glucose oxidation proceeded more quickly than in the other reactions; therefore, it was used to produce a large amount of the glucose oxidation product for NMR analysis. The reaction mixture with 10 mM guaiacol was composed of 20 mM sodium phosphate buffer (pH 8.0), 1 M glucose, 0.5 U/ml laccase as a guaiacol oxidase, and 60 U/ml *Cp*GDH.

### Thin-layer chromatography

A 1 μl portion of the solution containing the glucose oxidation product prepared with each mediator was applied onto the Silica gel 60 F254 plate (Merck) and dried. The plates were placed in a chromatography chamber and developed in a mobile phase (acetonitrile/water, 70/30) to detect glucose, 1,5-AG, aldehydes, and the products of their oxidation. The plate was then dried and either sprayed with 5% sulfate solution (sulfuric acid/ethanol, 5/95) or dipped in 1.4 mg/ml DNPH (FUJIFILM Wako Pure Chemical) solution (85% phosphoric acid/ethanol, 1/1) or BCG solution (BCG/ethanol/NaOH, 4 mg/100 ml/several drops). Subsequently, the plate was dried and heated to detect residual glucose, 1,5-AG, and the oxidation product of glucose and 1,5-AG. When a 5% sulfuric acid solution was applied, glucose, 1,5-AG, and glucuronic acid (TCI) were detected as black and brown spots. Gluconic acid (FUJIFILM Wako Pure Chemical) resulted in a smear spot. When the plate was dipped into the DNPH solution to demonstrate aldehyde production, the aldehyde was detected as a yellow spot if it was the product of substrate oxidation. However, as the yellow spots were difficult to visualize in photographs, we heated the TLC plate dipped in DNPH solution to change the color of the spots to blue. Acetonitrile, sulfuric acid, ethanol, phosphoric acid, and BCG were purchased from FUJIFILM Wako Pure Chemical.

### Detection of glucuronic acid and gluconic acid using assay kits

We demonstrated whether the product of glucose oxidation produced with *Cp*GDH was glucuronic acid or gluconic acid using the d-glucuronic/d-galacturonic acid assay kit (Megazyme) and F-kit (Roche), according to the manufacturer’s instructions. To prepare samples for evaluating the value of glucuronic acid, the reaction mixture was composed of 10 mM 1-m-PMS, 100 mM sodium phosphate buffer (pH 8.0), 50 mM glucose, 500 U/ml catalase, and 20 U/ml *Cp*GDH or *At*GDH. Crushed active carbon (Futamura Chemical) was added to the glucose oxidation product, and the mediator 1-m-PMS was removed; this is because electrons were transferred between the NAD included in the assay kits and reduced 1-m-PMS; therefore, we could not evaluate it reproducibly. When we prepared specimens for the gluconic acid assay, the reaction mixture was composed of 1.4 mM 1-m-PMS, 100 mM potassium phosphate buffer (pH 6.0), 500 mM glucose, 100 U/ml catalase, and 92 U/ml *Cp*GDH or 260 U/ml *At*GDH.

### HPLC

Glucuronic acid production by *Cp*GDH was demonstrated using an ABEE labeling kit (J-OIL MILLS) for the simultaneous detection of glucose and glucuronic acid. *Cp*GDH and other proteins were removed from the reaction mixture using an ultrafiltration membrane (10,000 molecular weight cutoff; Sartorius) before labeling glucose and the glucose oxidation product, followed by dilution with ultrapure water. The diluted specimens were then labeled according to the manufacturer's protocol, and contamination was removed using an ultrafiltration membrane. The labeled specimens were analyzed on an HPLC class VP series (Shimadzu) with two LC-10ADVP pumps, a system controller SCL-10AVP, CTO-10AVP column oven, and fluorescence detector RF-10AXL at excitation and emission wavelengths of 305 nm and 360 nm, respectively, using an HPLC Honenpak C18 column, 3 μm, 4.6 mm × 75 mm (J-OILS MILLS). The isocratic conditions used 100% solution A (0.02% trifluoroacetic acid/acetonitrile, 90/10) for 30 min at a flow rate of 1.0 ml/min.

Furthermore, we demonstrated that *Cp*GDH produces only glucuronic acid from glucose and does not produce gluconic acid by directly detecting them without using a labeling kit. For their simultaneous detection, the specimen was incubated for 5 min at room temperature in 3 mM perchloric acid, and 10 g/L 5-SA, *Cp*GDH, and other proteins were denatured and removed, followed by passing the specimen through a 0.45 μm filter. The specimens were analyzed by HPLC equipped with a refractive index detector, using the RSpak KC-811 column, 8.0 mm × 300 mm, and the guard column RSpak KC-G, 6.0 mm × 50 mm (Shodex). The isocratic conditions used a 3 mM perchloric acid solution at 40 °C for 20 min at a flow rate of 1.0 ml/min. Trifluoroacetic acid, perchloric acid, and 5-SA were purchased from FUJIFILM Wako Pure Chemical.

### Purification of the glucose oxidation product for MS and NMR

The glucose oxidation products of *Cp*GDH and guaiacol were separated using a Wakosil C18 column (FUJIFILM Wako Pure Chemical). The glucose oxidation product was applied to a column preequilibrated with ultrapure water and then eluted. The eluate was concentrated under reduced pressure. The concentrated specimen was mixed with ten times the volume of 99% ethanol and precipitated. The solid deposit was collected as a pellet by centrifugation and lyophilized using DRC-1000 (EYELA).

### Mass spectrometry

For the LC-MS system (Shimadzu) equipped with a COSMOSIL 5C18 AR-II column (4.6 × 150 mm, NACALAI TESQUE), mobile phase gradient elution was performed using 2.0 × 10^−2^% (v/v) acetic acid (elution A) and acetonitrile (elution B). The flow rate was 0.3 ml min^−1^. The gradient scheme of the analysis was as follows: 0 to 10 min, 0% of elution B; 10 to 25 min, linear gradient from 0 to 100% of elution B; 25 to 45 min, 100% of elution B; 45 to 55 min, linear gradient from 100 to 0% of elution B; and 55 to 60 min, 0% of elution B. The effluents were monitored using an SPD-M20A PDA and LCMS-2020 (Shimadzu). Detection was performed using negative-mode electrospray ionization. The MS conditions were as follows: interface temperature, 350 °C; desolvation line temperature, 250 °C; nebulizing gas flow, 1.5 L min^−1^; heat block temperature, 200 °C; and drying gas flow, 10 L min^−1^.

### NMR

We asked Honorary Professor Hiroki Hamada at the Okayama University of Science and lecturer Toshifumi Mizuta at Tottori University for ^1^H and ^13^C NMR analyses of the glucose oxidation product. The product was dissolved in D_2_O, and the chemical structures of the purified glucose oxidation product were determined using proton nuclear magnetic resonance (^1^H-NMR), carbon-13 nuclear magnetic resonance (^13^C-NMR), ^1^H-^1^H correlation spectroscopy, and ^1^H-detected multiple quantum coherence spectroscopy on an Avance II 600 (Bruker); 600 MHz and 150 MHz were used for ^1^H-NMR and ^13^C-NMR, respectively.

### Determination of the amount of DCIP needed for glucose oxidation

To determine the number of DCIP molecules required to oxidize one molecule of glucose to glucuronic acid, DCIP equivalent to three times the amount of glucose was added to the reaction mixture. Oxygen was removed from the reaction mixture by bubbling nitrogen through it for a few minutes before adding the enzyme solution. After complete glucose oxidation, the amount of reduced DCIP was calculated from the absorbance, indicating the amount of oxidized DCIP. The effect of dissolved oxygen on activity was evaluated using a microplate spectrophotometer. To determine the reaction ratio of glucose to DCIP, the spectrophotometer V-660 was used.

### Analysis of the amino acid sequence of *Cp*GDH and *Af*GDH

The amino acid sequences of *Af*GDH and *An*GOX registered in PDB database as 4YNU and GenBank as CAC12802.1 were used. The motif search was conducted by Motif search from GenomeNet. MAFFT was used for multiple alignment analysis and the alignment result was analyzed by ESPript3.0 (40).

### Preparation of the 3D structure model of *Cp*GDH

The 3D structure model of *Cp*GDH was built by SWISS-MODEL that is a fully automated protein structure homology-modeling server. To create a structure model of *Cp*GDH with FAD, the model of *Cp*GDH and the 3D structure of *Af*GDH (PDB ID: 4YNU) known as FAD-GDH were aligned in PyMOL (https://www.pymol.org/). We combined FAD of 4YNU with the *Cp*GDH model and output as a new PDB file. Their 3D structures were analyzed by PyMOL.

### Construction of a phylogenetic tree

A phylogenetic tree was constructed from the amino acid sequences of G6DHs, including *Cp*GDH. Other amino acid sequences were extracted from literature (41, 42, 43) and the CAZy family AA3, which includes enzymes that catalyze the oxidation of alcohols or carbohydrates. Based on their amino acid sequences, we conducted sequence alignment and constructed a phylogenetic tree using MEGA X software (https://www.megasoftware.net/) (44, 45). The neighbor-joining method of MEGA X was used to construct a phylogenetic tree.

## Data availability

All data are available in the manuscript and Supporting Information. Nucleotide sequence data are available in the DDBJ under accession numbers LC739014, *Cp*GDH; LC739015, *Gs*GDH; LC739016, *Fla*GDH; LC739017, *As*GDH; LC739018, *Ds*GDH; LC739019, *Pc*GDH; LC739020, *Ko*GDH; LC739021, *Gsp*GDH; LC740184, *Cglo*GDH; LC740185, *Cgo*GDH; LC740186, *Co*GDH; and LC740187, *Cta*GDH.

## Supporting information

This article contains Supporting information (46, 47, 48, 49, 50, 51, 52, 53, 54).

## Conflict of interest

This research was conducted with research funding from Ikeda Food Research Co., Ltd, to which the primary and secondary authors belong. The primary and second authors are employees of Ikeda Food Research Co., Ltd and received compensation from Ikeda Food Research Co, Ltd. The other authors declare that they have no conflicts of interest with the contents of this article.
