# Supplementary material for: Discovery and characterization of an FAD-dependent glucose 6-dehydrogenase
Source: J Biol Chem. 2025 Jan 13;301(3):108189. doi: 10.1016/j.jbc.2025.108189 (PMC11871447; doi:10.1016/j.jbc.2025.108189)
Supplement: Supplementary information [file mmc1.docx]

**SUPPORTING INFORMATION**

**Discovery and characterization of an FAD-dependent glucose 6-dehydrogenase**

Takahiro Fujii^1^, Michinari Honda^1^, Wataru Fujii^2^, Yoshimi Shimada^2^, Michiki Takeuchi^3,4^, and Jun Ogawa^2, *^

***Materials***

Potassium phosphate buffer prepared with potassium dihydrogen phosphate and dipotassium hydrogen phosphate, 1-m-PMS, DCIP, substrates (d-glucose, 1,5-AG, cellobiose, 2-deoxy-d-glucose, *N*-acetyl-d-glucosamine, l-sorbose, d-mannose, d-fructose, trehalose, d-glucosamine, d-sorbitol, d-xylose, d-glucose 6-phosphate, d-arabinose, l-arabinose, d-fucose, l-fucose, d-galactose, l-glucose, l-gulose, d-lyxose, l-lyxose, l-mannose, d-raffinose, l-rhamnose, d-tagatose, glucuronic acid sodium salt, lactose, maltose, sucrose, d-mannitol, methanol, ethanol, and glycerol), Fehling solution composed of copper (II) sulfate and potassium sodium (+)-tartrate tetrahydrate, TBHQ, purified *At*GDH, purified G6DHs (*Cp*GDH, *Cglo*GDH, *Cgo*GDH, *Co*GDH, *Csp*GDH, *Gs*GDH, *Gsp*GDH, *As*GDH, *Cta*GDH, *Ds*GDH, *Fla*GDH, *Ko*GDH, and *Pc*GDH), and catalase.

**Supporting Table 1.**

**Fehling’s test.** Fehling’s test was used to confirm that *Cp*GDH did not oxidize the C1-position of sugars. Fehling's solution was prepared by mixing equal volumes of 0.28 M copper (II) sulfate solution and 1.2 M potassium sodium (+)-tartrate tetrahydrate solution. The d-glucose oxidation product prepared with *At*GDH, indicated as G1DH, which oxidizes the C1-position of sugars, such as glucose, and produces gluconolactone, did not give a red precipitate. In contrast, the sugar oxidation products prepared with *Cp*GDH produced a red precipitate, suggesting that the hydroxyl group at the reducing end was retained.

| Sample | Result | Remark |
| --- | --- | --- |
| Buffer + TBHQ | Negative | Negative control |
| Glucose | Positive | Positive control |
| Oxidized product of glucose  (gluconic acid) | Negative | Prepared with *At*GDH |
| Oxidized product of glucose (glucuronic acid) | Positive | Prepared with *Cp*GDH |
| Cellobiose | Positive | - |
| Oxidized product of cellobiose | Positive | Prepared with *Cp*GDH |
| 2-Deoxy-glucose | Positive | - |
| Oxidized product of 2-deoxy-glucose | Positive | Prepared with *Cp*GDH |
| GlcNAc | Positive | - |
| Oxidized product of GlcNAc | Positive | Prepared with *Cp*GDH |
| Mannose | Positive | - |
| Oxidized product of mannose | Positive | Prepared with *Cp*GDH |
| Negative, no precipitate was detected. | |  |
| Positive, a red precipitate was produced. | |  |

**Supporting Figure 1.**

**TLC of the glucose oxidation products prepared using various G6DHs.** New sequences encoding G6DHs were identified using BLASTP, based on the amino acid sequence of *Cp*GDH. Plasmids with these sequences inserted were used to generate *Aspergillus oryzae* transformants, and the expressed proteins were purified. To evaluate the oxidation of glucose to glucuronic acid, glucose oxidation products were prepared using a reactive mixture consisting of 10 mM 1-m-PMS, 100 mM potassium phosphate buffer (pH 7.0), 50 mM glucose, 500 U/mL catalase, and each of the new G6DHs. The TLC results indicated that 12 GDHs oxidized glucose to glucuronic acid, and they were G6DHs similar to *Cp*GDH. The enzymes included *Cglo*GDH from *Colletotrichum gloeosporioides*, *Cgo*GDH from *Colletotrichum godetiae*, *Co*GDH from *Colletotrichum orbiculare*, *Csp*GDH from *Colletotrichum* sp., *Gs*GDH from *Glomerella* sp., *Gsp*GDH from *Gaeumannomyces* sp., *As*GDH from *Acremonium strictum*, *Cta*GDH from *Colletotrichum tanaceti*, *Ds*GDH from *Diaporthaceae* sp., *Fla*GDH from *Fusarium langsethiae*, *Ko*GDH from *Khuskia oryzae*, and *Pc*GDH from *Phialemoniopsis curvata*. Glc, d-glucose; GlcA, glucuronic acid.

**Supporting Figure 2.**

**Characterization of *Colletotrichum plurivorum* glucose dehydrogenase (*Cp*GDH).** (A) Spectra of GDHs. Blue solid line, only *Aspergillus terreus* GDH (*At*GDH); green solid line, only *Cp*GDH; red dashed line, *At*GDH with glucose; blue dot line, *Cp*GDH with glucose. (B) pH stability of *Cp*GDH. The buffers potassium acetate, McIlvaine, potassium phosphate, Tris-HCl, and glycine-NaOH are represented by red circle, blue square, green triangle, purple diamond, and orange square, respectively. (C) Thermal stability of *Cp*GDH. (D) Inhibitor of *Cp*GDH. TritonX-100, Hydrogen peroxide, FeCl_3_, and CuCl_2_ were used as inhibitors and shown by red circle, blue square, green triangle, and purple diamond, respectively.

**Supporting Table 2.**

**Characteristics and kinetic parameters of G6DHs.** The properties and kinetic parameters of the purified G6DHs were evaluated similar to those of *Cp*GDH. The substrate was glucose, while DCIP and 1-m-PMS were used as mediators. A microplate spectrophotometer was used for the assays. Relative activity was defined as the ratio of enzyme activity towards glucose and that towards other substrates; the enzyme activity towards glucose in each specimen was taken as 100.

|  | Specific activity | *K*_m_ | Relative  activity (%) | | Stability | |
| --- | --- | --- | --- | --- | --- | --- |
|  | (U/mg) | (mM) | Mal | Xyl | pH | Temp (°C) |
| *As*GDH | 32.7 | 42.4 | 0.5 | 0.2 | 5.0–9.6 | 50 |
| *Cgo*GDH | 102 | 307 | 0.2 | 0.4 | 4.4–9.3 | 40 |
| *Cglo*GDH | 44.5 | 573 | 0.1 | 0.4 | 5.5–9.3 | 40 |
| *Co*GDH | 39.3 | 441 | 0.2 | 0.5 | 4.4–9.3 | 45 |
| *Csp*GDH | 109 | 40.2 | 0.2 | 0.6 | 3.2–9.3 | 45 |
| *Cta*GDH | 84.3 | 167 | 0.2 | 0.1 | 5.6–9.7 | 50 |
| *Ds*GDH | 26.9 | 215 | 0.2 | 0.1 | 4.9–8.7 | 40 |
| *Fla*GDH | 18.2 | 1,120 | 0.1 | 0.1 | 4.4–9.7 | 45 |
| *Gs*GDH | 43.4 | 425 | 0.2 | 0.4 | 5.0–9.3 | 40 |
| *Gsp*GDH | 40.8 | 42.2 | 0.3 | 0.5 | 5.5–8.7 | 40 |
| *Ko*GDH | 12.2 | 40.4 | 0.4 | 0.2 | 5.5–8.7 | 35 |
| *Pc*GDH | 2.65 | ND^a^ | 0.4 | ND | 4.1–9.6 | 50 |

ND^a^, not detected

**Supporting Table 3.**

**Substrate specificity**. A reaction mixture comprising 100 mM substrate, 33 mM potassium phosphate buffer (pH 6.0), 0.2 mM 1-m-PMS, and 0.14 mM DCIP was prepared for the activity assay, using an absorption coefficient of 10.8 mM^−1^cm^−1^ at 600 nm. Relative activity was defined as the ratio of enzyme activity towards glucose and that towards other substrates; the activity towards glucose in each specimen was taken as 100.

|  | Concentration | Relative activity (%) | |
| --- | --- | --- | --- |
| Substrate | (mM) | *Cp*GDH | *At*GDH |
| d-Glucose | 100 | 100 | 100 |
| 1,5-AG | 100 | 108 | 1 |
| Cellobiose | 100 | 83 | ND^a^ |
| 2-Deoxy-d-glucose | 100 | 70 | 28 |
| *N*-Acetyl-d-glucosamine | 100 | 32 | ND |
| l-Sorbose | 100 | 17 | ND |
| d-Mannose | 100 | 7 | 1 |
| d-Fructose | 100 | 6 | ND |
| Trehalose | 100 | 2 | 2 |
| d-Glucosamine | 100 | 2 | ND |
| d-Sorbitol | 100 | 1 | ND |
| d-Xylose | 100 | ND | 12 |
| d-Glucose 6-phosphate | 100 | ND | 1 |
| d-Arabinose | 100 | ND | ND |
| l-Arabinose | 100 | ND | ND |
| d-Fucose | 100 | ND | ND |
| l-Fucose | 100 | ND | ND |
| d-Galactose | 100 | ND | ND |
| l-Glucose | 100 | ND | ND |
| l-Gulose | 100 | ND | ND |
| d-Lyxose | 100 | ND | ND |
| l-Mannose | 100 | ND | ND |
| d-Raffinose | 100 | ND | ND |
| l-Rhamnose | 100 | ND | ND |
| d-Tagatose | 100 | ND | ND |
| Glucuronic acid sodium salt | 100 | ND | ND |
| Lactose | 100 | ND | ND |
| Maltose | 100 | ND | ND |
| Sucrose | 100 | ND | ND |
| d-Mannitol | 100 | ND | ND |
| Methanol | 100 | ND | ND |
| Ethanol | 100 | ND | ND |
| Glycerol | 100 | ND | ND |

ND^a^, not detected

**Supporting Table 4.**

**Mediator reactivity**. The reaction mixture comprised 100 mM potassium phosphate buffer (pH 7.0) or 100 mM sodium phosphate buffer (pH 8.0), 50 mM glucose, 10 mM mediator, 500 U/mL catalase, and 20 U/mL *Cp*GDH. For reactions that did not proceed after mixing, laccase was added. Glucose was oxidized with *Cp*GDH overnight at 25 ºC, and the glucose oxidation product was then developed using TLC. When a spot of glucose disappeared or the glucose oxidation product was detected, the reaction was considered positive. Redox potentials were obtained from previous studies (46–54).

| Mediator | Reactivity | *E*°´ (vs NHE) |
| --- | --- | --- |
| NADP^+^ | − | −0.324 |
| NAD^+^ | − | −0.320 |
| FAD | − | −0.219 |
| FMN | − | −0.219 |
| Vitamin B_2_ | − | −0.200 |
| Vitamin K_3_ | − | 0.009 |
| Methylene blue | + | 0.011 |
| 1,4-Naphthoquinone | + | 0.036 |
| Thionine | − | 0.064 |
| Ascorbic acid | − | 0.080 |
| PMS | + | 0.080 |
| 1-m-PMS | + | 0.168 |
| DCIP | + | 0.217 |
| Cytochrome c | − | 0.254 |
| Benzoquinone | + | 0.280 |
| Hydroquinone | + | 0.293 |
| *tert*-Butylhydroquinone | + | 0.300 |
| Catechol | + | 0.301 |
| Potassium ferricyanide | + | 0.430 |
| ABTS | + | 0.670 |

***References***

46. Fultz, M., and Durst, R. (1982) Mediator compounds for the electrochemical study of biological redox systems: a compilation. *Anal. Chim. Acta* **140**, 1-18

47. Giraud, W., Mirabel, M., and Comtat, M. (2013) Electroanalysis may be used in the vanillin biotechnological production. *Appl. Biochem. Biotechnol.* **172**, 1953-1963

48. Hickey, D., Milton, R., Rasmussen, M., Abdellaoui, S., Nguyen, K., and Minteer, S. (2016) Fundamentals and applications of bioelectrocatalysis. *Electrochemistry* **13**, 97-132

49. Kano, K. (2022) Fundamental insight into redox enzyme-based bioelectrocatalysis. *Biosci. Biotechnol. Biochem.* **86**, 141-156

50. Lundblad, R. L., and Macdonald, F. (2010) *Handbook of Biochemistry and Molecular Biology*, 4th Ed., CRC Press, Boca Raton

51. Motin, M. A., Uddin, M. N., Dhar, P., Mia, M. A., and Hashem, M. A. (2016) Voltammetric electro-synthesis of catechol-aspartic acid adduct at different pHs and concentrations. *Anal. Bioanal. Electrochem.* **8**, 505-521

52. Osakai, T., Kuwabata, S., and Kano, K. (2000) *Basic Electrochemistry*, 1st Ed., Kagaku-Dojin, Japan

53. Schafer, F. Q., and Buettner, G. R. (2003) Redox State and Redox Environment in Biology, in *Signal Transduction by Reactive Oxygen and Nitrogen Species: Pathways and Chemical Principles* (Forman, H. J., Fukuto, J., and Torres, M. eds.), Springer Netherlands, Dordrecht. pp 1-14

54. Vasdev, S., Gill, V., and Singal, P. (2006) Modulation of oxidative stress-induced changes in hypertension and atherosclerosis by antioxidants. *Exp. Clin. Cardiol.* **11**, 206-216
